# TLC-Based Metabolite Profiling and Bioactivity-Based Scientific Validation for Use of Water Extracts in AYUSH Formulations

**DOI:** 10.1155/2021/2847440

**Published:** 2021-12-31

**Authors:** Sultan Zahiruddin, Abida Parveen, Washim Khan, Rabea Parveen, Sayeed Ahmad

**Affiliations:** ^1^Bioactive Natural Product Laboratory, Department of Pharmacognosy and Phytochemistry, School of Pharmaceutical Education and Research, Jamia Hamdard, New Delhi 110062, India; ^2^Department of Clinical Research, School of Interdisciplinary Sciences and Technology, Jamia Hamdard, New Delhi 110062, India; ^3^National Center for Natural Products Research, School of Pharmacy, University of Mississippi, University, Oxford, MS 38677, USA

## Abstract

We aimed to develop a chromatographic method for scientific validation of water extract of some important Indian traditional plants used in AYUSH-based formulation as immunomodulator and to evaluate their bioactive potential. Fruits of *Phyllanthus emblica* L. and *Piper nigrum* L., stem of *Tinospora cordifolia* (Willd.) Miers, rhizome of *Curcuma longa* L., leaves of *Ocimum sanctum* L. and *Achillea millefolium* L., roots of *Withania somnifera* L., and stem bark of *Azadirachta indica* A. Juss. were coarsely powdered and extracted in three different solvents (water, ethanol, and hydroethanol). The antioxidant potential was determined through 1, 1-diphenyl-2-picrylhydrazyl and ferric reducing capacity methods. Thin-layer chromatography (TLC) was carried out for the comparative metabolite profiling of the extracts using toluene, ethyl acetate, and formic acid (5 : 4 : 1, v/v/v) as a solvent system. *In vitro* immunomodulatory activity of the extracts has been tested on splenocyte proliferation and pinocytic assay. Hydroethanolic extract (HEE) of most of the plant materials has the highest phenolic and flavonoid contents, followed by water extract (WE) and ethanolic extract (EE), whereas the water extracts of most of the plant material showed better antioxidant activity. Almost all extract exhibited splenocyte proliferation and pinocytic activity in a dose-dependent manner. But water extract showed significantly higher splenocyte proliferation and pinocytic activity as compared to the other two extracts. TLC analysis resulted in detection of totally 63 and 56 metabolites at 254 nm and 366 nm, respectively. Through principal component analysis (PCA), it was observed that metabolite pattern of different extracts from same plant materials may be different or similar. This preliminary result can be used for quality evaluation and to develop a synergy-based polyherbal combination of water extracts of selected plant materials.

## 1. Introduction

Herbs and plants not only provide nutritional benefit but also help prevent and manage different diseases. According to World Health Organization, about 80% of the world population trust on conventional remedies for some part of primary health care [1]. The majority of India's population uses herbs for basic healthcare needs. India is found to have diverse and a vast collection of medicinal plants. In India, treatment with herbs becomes an alternative way to cure the patients, and since historical times, this approach is in practice [2].

In India, there are six traditional systems (namely Ayurveda, Yoga & Naturopathy, Unani, Siddha, and Homoeopathy) being followed for preventing and managing disease. These systems are collectively called as AYUSH. It includes various plants with different ethnopharmacological relevance such as immunostimulant, antibacterial, antiviral, anticancer, and adaptogenic. In AYUSH, more than twenty plant materials have been recommended as immunomodulators and most of them have been separately proved for their immune-enhancing impact on humans [2, 3]. Some important plants are *Phyllanthus emblica, Piper nigrum, Tinospora cordifolia, Curcuma longa, Withania somnifera, Ocimum sanctum, Achillea millefolium, Azadirachta indica,* etc.

Because herbal formulation is becoming more popular, accurate scientific data are essential to define the quality of these plant materials. Chromatographic fingerprinting has become a significant quality control method for herbal samples and extracts. A plant material's chromatographic fingerprint is a chromatographic pattern of the extract of some similar active compounds with pharmacologically active and/or chemical properties. The WHO has approved fingerprint analysis as a tool for ensuring the quality of herbal samples and extracts [4]. Constituents inside herbal samples that can be utilized to validate their potency or identification are referred to as marker compounds. The marker compounds are frequently referred to as active components that confirm the starting material's identity. Some traditionally used plant materials have unknown active marker compounds, and others have several marker compounds. Identifying accurate marker compounds for all traditionally used plant materials is tremendously difficult. Even if the quantity of active constituents is not precisely the same for different samples, TLC fingerprints can accurately authenticate and identify the plant materials. As a result, obtaining reliable TLC fingerprints that represent the plant materials' bioactive compounds and chemically distinctive components is essential [5–7].

TLC fingerprint of plant extracts is becoming a routine analytical method because of its low operational cost, high sample throughput, and minimum sample cleanup. TLC has various advantages, including running multiple samples simultaneously with a little amount of mobile phase and lowered analysis time and cost per sample [8, 9].

Our study has screened eight Indian medicinal plant materials for their quality evaluation and immunomodulatory activity. Fruits of *P. emblica* and *P. nigrum*, stem of *T. cordifolia*, rhizome of *C. longa,* leaves of *O. sanctum* and *A. millefolium,* roots of *W. somnifera,* and stem bark of *A. indica* were screened. The metabolite profiling was performed using TLC. In addition to metabolite profiling, a new statistical method was employed to build scientific correlations between the metabolites of extracts and plant materials. These plant materials have been used in several traditional formulations for various types of diseases.

## 2. Material and Methods

### 2.1. Chemicals

3-(4, 5-Dimethylthiazol-2-yl)-2, 5-diphenyltetrazolium bromide (MTT), neutral red, 2, 2-diphenyl-1-picrylhydrazyl (DPPH), and Folin–Ciocalteu were purchased from Sisco Research Laboratories Pvt. Ltd. (SRL, India). RPMI 1640 medium and fetal bovine serum (FBS) were purchased from HiMedia Laboratories Pvt. Ltd. (India). Aluminum chloride was purchased from S D Fine-Chem Limited (Mumbai, India). All the other chemicals and reagents used were of analytical grade and were checked to ensure they were not expired before the experiment.

### 2.2. Collection of Plant Material

Fruits of *P. emblica* and *P. nigrum*, leaves of *O. sanctum* and *A. millefolium,* stem of *T. cordifolia*, roots of *W. somnifera*, rhizomes of *C. longa,* and stem bark of *A. indica* were purchased from Universal Biotech (Delhi, India). These purchased plant materials were further authenticated through phytochemical and physiochemical evaluation as per protocol mentioned in Indian Pharmacopoeia [10], Ayurvedic Pharmacopoeia of India [11]. A voucher specimen of each plant material has been deposited in our laboratory depository for future reference. Samples were coarsely powdered, covered with aluminum foil (to avoid further exposure of light and moisture), and stored until experimental use.

### 2.3. Extract Preparation

The course powder of each plant materials was divided into three equal parts. Each part was macerated for 24 h using water, ethanol, and 50% ethanol (hydroethanol) separately. After maceration, ethanolic extract (EE) and hydroethanolic extract (HEE) was further refluxed for 3 h but not for water extract (WE). All these three extracts were filtered, and the filtrate was evaporated to dryness under reduced pressure. The percentage yields of different extracts were calculated, and dried extracts were stored at 4°C for further analysis.

### 2.4. Estimation of Total Phenolic and Flavonoid Contents

Total phenolic content (TPC) and total flavonoid content (TFC) in the extract were determined as previously reported protocol [12]. For TPC determination, 500 *μ*L of the extract (5 mg/mL) was mixed thoroughly with 2.5 mL of Folin–Ciocalteu reagent and 2.5 mL of sodium carbonate (75 g/L) followed by incubation in the dark for 30 min. After incubation, optical density was recorded at 760 nm against a blank. TPC was expressed as milligram of gallic acid equivalent per gram dry weight of the extract.

For the determination of TFC, 500 *μ*L of the extract (5 mg/mL) was added in 0.1 mL of aluminum chloride (10% w/v) and 0.1 mL potassium acetate (0.1 mM). The resulting mixture solution made up to 10 mL with distilled water, the mixture was kept at room temperature for 30 min, and absorbance was recorded at 415 nm. TFC was expressed as milligram of quercetin equivalent per gram dry weight of the extract.

### 2.5. Antioxidant Activity of the Extracts

The antioxidant activity of each extract was expressed as DPPH scavenging potential (DPPH) and ferric reducing capacity. For the DPPH scavenging assay, 1 mL of freshly prepared DPPH solution (0.3 mM in methanol) was mixed with 1 mL of extract (10–500 *μ*g/mL) and tubes were allowed to stand for 25 min. The reaction control was prepared as above without having any extract. After incubation, the absorbance was measured at 515 nm. The half-maximal inhibitory concentration (IC_50_) was used to measure radical scavenging activity. The ability of the sample to scavenge the DPPH radicals was calculated using the following formula:

(1) $$\begin{array}{c} \text{DPPH radical scavenging effect}=\frac{\left(A_{\text{control}}-A_{\text{sample}}\right)\times 100}{A_{\text{control}}}. \end{array}$$

The absorbance of the DPPH solution without extract is *A*_control_, and the absorbance of the sample with the DPPH solution is *A*_sample_.

To determine the ferric reducing capacity, the extract of varying concentrations (10–500 *μ*g/mL) was taken in 2.5 mL of phosphate buffer (pH 6.6) in a test tube, and 2.5 mL of potassium ferricyanide solution (1% w/v) was added. The mixture was then incubated in a water bath at 50°C for 30 min. After the incubation, 2.5 mL of trichloroacetic acid (10% w/v) was added and centrifuged. The supernatant (2.5 mL) was diluted with an equal amount of distilled water, and freshly prepared 0.5 mL ferric chloride (0.1% w/v) was added. Then, the mixture was mixed thoroughly, and its absorbance was measured at 700 nm.

### 2.6. Comparative Metabolite Profiling of the Extracts by TLC

Ten milligrams of each extract was dissolved in one milliliter of HPLC grade methanol separately to obtain 10 mg/mL working stock solutions. Stock solutions were then filtered, and 5 *μ*L of each extract solution was separately applied on silica gel 60 F254 precoated TLC plates, 20 × 10 cm (Merck, Germany) with the help of Camag Linomat V (Camag, Switzerland) applicator. The sample solution was applied to a 6 mm wide band using Camag Linomat V automated TLC applicator with the nitrogen flow providing a delivery speed of 120 nL/s from the syringe. Applied plates were presaturated with the mobile phase for 30 min in a Camag twin through glass tank and allowed to move the analytes in Camag horizontal developing chamber (20 × 10) at a room temperature (25°C) using solvent system toluene: ethyl acetate: formic acid (5 : 4 : 1, v/v/v). After the solvent run up to 80% of total height, the plate was air-dried. Some phenolic UV compounds showed absorption maxima at long-wave UV [13]. To get the optimum intensity of each compound, developed TLC plates were scanned at both 254 nm (short-wave UV) and 366 nm (long-wave UV) by a Camag TLC scanner III using the WinCATS software.

### 2.7. Metabolite Comparison of TLC Detected Compounds

Compounds detected through TLC of each extract were further processed for metabolite comparison. The presence (area of detected compound in TLC plate) or absence (Value 0) of the compound in different extracts was used to normalize the data. For multivariate data analysis and principal component analysis (PCA), the normalized data were further processed through XLSTAT 2014.5.03. The extended statistics (XS) module of the XLSTAT program was used for orthogonal projections to latent structures discriminant analysis (OPLS-DA) of metabolic data collected from the TLC dataset. To select major constituents, OPLS-DA with Pareto scaling was used to find metabolite variations that are responsible for separation among the different extracts. The heatmap was implemented using the MetaboAnalyst program for unsupervised clustering based on the major metabolites collected with OPLS-DA. We separated the different extracts for eight different plant materials using a MetaboAnalyst data annotation methodology. The network intersection was determined to conduct a comparative study based on constituent patterns in different extracts of eight different plant materials. The Ward distance algorithm was used to calculate the distance between the different generated clusters for hierarchical cluster study (HCA) analysis [12, 14].

### 2.8. Quantitative Analysis of Polyphenolic Compounds from Water Extracts

Gallic acid, quercetin, and ferulic acid were selected as common polyphenolic marker compounds for all plant materials. These markers were quantified using high-performance thin-layer chromatography. Twenty milligrams of each extract were dissolved separately in HPLC grade methanol to get 20 mg/mL solutions. The stock solutions of standard gallic acid, quercetin, and ferulic acid (Sigma-Aldrich) were prepared in HPLC grade methanol to get a concentration of 1000 *μ*g/mL. Mix all the stock solution, and get a concentration of 333.33 *μ*g/mL. The prepared samples were filtered using a 0.2 *μ*m PTFE membrane filter before analysis. The prepared extracts and standards were separately applied on silica gel 60 F254 precoated TLC plates, 20 × 10 cm (Merck, Germany) with the help of Camag Linomat (Camag, Switzerland) applicator with nitrogen flow providing a delivery speed of 120 nL/s from the syringe. Toluene: ethyl acetate: formic acid (5 : 4 : 1; v/v/v) were used as developing solvents for gallic acid, quercetin, and ferulic acid. Plates were developed horizontally in a Camag twin trough glass chamber (20 × 10 cm), presaturated with the mobile phase for 30 min. The developed plates were air-dried and scanned at 254 nm by Camag TLC densitometric scanner III operated by WinCATS software.

Piperine, berberine, withaferin A, and curcumin were selected as specific marker compounds for fruits of *P. nigrum*, stem of *T. cordifolia*, roots of *W. somnifera,* and rhizome of *Curcuma longa,* respectively. Ten milligrams of the each water extract was dissolved separately in HPLC grade methanol to get 10 mg/mL solutions. The stock solutions of standard piperine, berberine, withaferin A, and curcumin (Sigma-Aldrich) were prepared in HPLC grade methanol to get a concentration of 500 *μ*g/mL. The prepared samples were filtered using a 0.2 *μ*m PTFE membrane filter before analysis. The extracts and standards were separately applied on silica gel 60 F254 precoated TLC plates, 10 × 10 cm (Merck, Germany) with the help of Camag Linomat (Camag, Switzerland) applicator with nitrogen flow providing a delivery speed of 120 nL/s from the syringe. Toluene: ethyl acetate: formic acid (5 : 4:1; v/v/v) were used as developing solvents for piperine, withaferin A, and curcumin, while *n*-butanol: water: acetic acid (4 : 5:1; v/v/v) were used for berberine. Plates were developed horizontally in a Camag twin trough glass chamber, presaturated with the mobile phase for 30 min. The developed plates were air-dried and scanned by Camag TLC densitometric scanner III operated by WinCATS software. Piperine was scanned at 254 nm, while berberine was scanned at 366 nm. In comparison, curcumin was scanned at 420 nm. The air-dried plate was derivatized in a 5% anisaldehyde sulfuric acid solution and scanned at 540 nm for quantitative analysis of withaferin A.

### 2.9. In vitro Immunomodulatory Activity

*In vitro* immunomodulatory activity of each extract was determined through splenocyte proliferation and pinocytic activity assay. Spleen cell and pinocytes were collected from adult BALB/c mice (6 weeks old, 25 ± 5 g). Mice were obtained from Central Animal House Facility of Jamia Hamdard (Registration No 173/GO/RE/S/2000/CPCSEA). Before initiation of the experiment, all experimental protocols were approved by the Institutional Animal Ethics Committee, and experiments were strictly performed according to the guidelines of the CPCSEA, New Delhi, India (Animal Approval Number 1551).

#### 2.9.1. Spleen Cell Proliferation Assay

Mice were placed into a chamber filled with the vapor of the isoflurane (anesthetic), and after 2 minutes, respiration of mice was ceased. The spleen of mice was removed, cut into small pieces, and homogenized with phosphate buffer solution (PBS) to obtain a suspension of spleen cells. It was then centrifuged, and cells were separated. Four milliliters of Tris-HCl-NH_4_Cl (pH 7.2) was added to 500 *μ*L of red spleen cells. The cell suspension was diluted fivefold with RPMI medium (RPMI-1640, Millipore Sigma, India) and centrifuged, and the cell pellet was resuspended in RPMI medium to get four million cells/mL. An aliquot of 100 *μ*L of cell suspension and 25 *μ*L of mitogen Con A was added to a 96-well plate. Each extract of 25 *μ*L was added to the above cells in the presence of Con A and incubated at 37°C in a CO_2_ incubator for 72 h. After incubation, 20 *μ*L of tetrazolium dye MTT 3-(4, 5-dimethylthiazol-2-yl)-2, 5-diphenyltetrazolium bromide was added to each plate and incubated for another 6 h. In a microplate reader, the optical density was measured at 490 nm [15]. The extract was treated only by RPMI medium for the negative control and results were expressed compared to the negative control.

#### 2.9.2. Pinocytic Activity Assay

Thioglycolate (3 mL of 1 mg/mL in PBS) was injected intraperitoneally into mice three days before sterile peritoneal lavage. Through peritoneal lavage with 10 mL of PBS, peritoneal macrophages were collected. The isolated cells were seeded and cultured in an RPMI medium containing 10% FBS. The collected cells (200 *μ*L) were transferred into 96-well plates and incubated for 4 h to adhere to the wall of the plate. The pinocytic activity was measured by neutral red uptake. Each extract of 25 *μ*L was added and incubated for 48 h. After incubation, 100 *μ*L of neutral red solution (0.1% in 10 mM PBS) was added and incubated for another 2 h. Discard supernatant with PBS to remove neutral red. Then, 100 *μ*L of neutral red detainer (ethanol and acetic acid (0.1% in water) in a ratio of 1 : 1, v/v) was added. The optical density was measured at 540 nm [15]. For negative control, RPMI medium was used in place of an extract, and results were expressed compared to the negative control.

## 3. Results

The authenticated plant material was extracted in three different solvents (water, ethanol, and hydroethanol) by overnight maceration and followed by reflux. The percentage yield of all extracts was calculated and is shown in Table 1.

### 3.1. Phenolic and Flavonoid Contents of the Extracts

Phenolic compounds are the secondary metabolites that plants might produce in response to foreign materials. These metabolites can neutralize the free radicals by their multifunctional properties by hydrogen donating, reducing, and singlet oxygen quenching properties. In our study, TPC in hydroethanolic extract (HEE) was highest, followed by water (WE) and ethanolic extract (EE). Among all three extracts of eight different plant materials, maximum phenolic content was found in *P. emblica* (42.35 ± 1.70%) followed by *W. somnifera* (38.42 ± 1.54%), *A. indica* (34.46 ± 2.99%), *O. sanctum* (26.48 ± 1.55%), *P. nigrum* (20.38 ± 2.16%), *A. millefolium* (19.51 ± 2.18%), *T. cordifolia* (19.30 ± 1.24%), and *C. longa* (12.78 ± 1.10%). HEE of each plant materials has the highest TPC as compared to WE and EE. The percentage of TPC of all other extracts of different plant materials is shown in Table 2.

The flavonoids are a large group of naturally occurring phenylchromones found in different parts of plant materials. TFC was found highest in HEE, similar to TPC. Among all three extracts of plant materials, the highest flavonoid content was found in *P. emblica* (20.15 ± 1.22%) followed by *W. somnifera* (19.58 ± 1.58%), *O. sanctum* (18.08 ± 2.62%), *A. indica* (17.64 ± 1.15%), *T. cordifolia* (14.85 ± 1.88%), *C. longa* (13.77 ± 1.30%), *P. nigrum* (11.56 ± 1.49%), and *A. millefolium* (10.24 ± 0.81%).The percentage of TFC of all other extracts of different plant materials is shown in Table 2.

### 3.2. Antioxidant Potential of the Extracts

The antioxidant potential of the extracts of plant materials is shown in Table 3. In the DPPH method, the degree of discoloration indicates the scavenging activity of an antioxidant compound. The best DPPH radical scavenging potential was found in water extracts of all plant material except the *A. millefolium*. The IC_50_ of DPPH free radical scavenging ability of all plant material is shown in Table 3, and data were compared with the antioxidant activity of ascorbic acid. The IC_50_ of a compound is inversely proportional to its antioxidant activity. The best DPPH radical scavenging potential was found for water extract of *W. somnifera* (IC_50_ : 85.96 ± 3.42 *μ*g/mL) followed by *P. emblica* (IC_50_ : 97.26 ± 1.14), *T. cordifolia* (IC_50_ : 105.65 ± 3.33), *P. nigrum* (IC_50_ : 112.18 ± 2.11), *A. millefolium* (IC_50_ : 145.56 ± 2.22), *A. indica* (IC_50_ : 156.83 ± 0.88), *C. longa* (IC_50_ : 199.15 ± 0.42), and *O. sanctum* (IC_50_ : 201.56 ± 1.11). The IC_50_ of all other extracts of different plant materials is shown in Table 3. In *W. somnifera* and *P. emblica*, all three extracts showed potent free radical scavenging activity.

Reducing power of the extract from ferricyanide into ferrocyanide was conducted on all the plant extracts to confirm its antioxidant potential. The reducing power of the different extracts of plant material is shown in Table 3. The highest reducing power potential was found similar in the WE of *W. somnifera* and *P. emblica* at 100 *μ*g/mL, equivalent to 80 *μ*g/mL of ascorbic acid. Reducing power studies suggested that almost the WE of all plant material possessed good reducing power compared to all the other extracts of plant material. The reducing power potential of plant material was linearly proportionate to the radical scavenging activity of DPPH.

### 3.3. Comparative Metabolite Profiling of the Extracts

**Figure 1:**
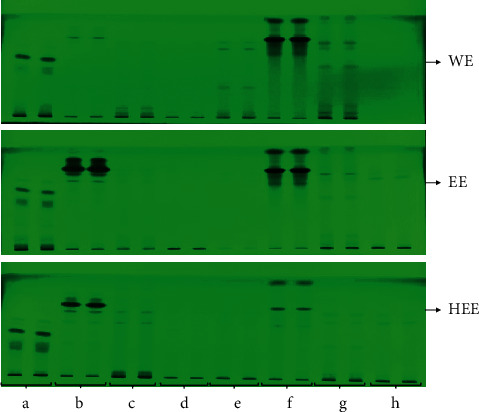
Developed TLC plate photograph of WE (water extract), EE (ethanolic extract), and HEE (hydroethanolic extract) of plant materials at 254 nm. (a) *P. emblica*; (b) *P. nigrum*; (c) *T. cordifolia*; (d) *W. somnifera*; (e) *A. indica*; (f) *C. longa*; (g) *O. sanctum*; (h) *A. millefolium*.

**Figure 2:**
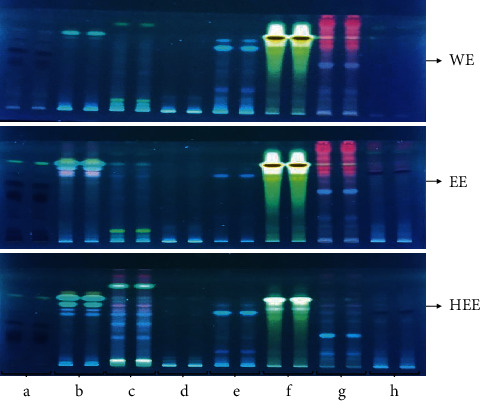
Developed TLC plate photograph of WE (water extract), EE (ethanolic extract), and HEE (hydroethanolic extract) of plant materials at 366 nm. (a) *P. emblica*; (b) *P. nigrum*; (c) *T. cordifolia*; (d) *W. somnifera*; (e) *A. indica*; (f) *C. longa*; (g) *O. sanctum*; (h) *A. millefolium*.

Thin-layer chromatography was used to separate the metabolites of various extracts of eight different plant materials. Various mobile phases with varied ratios were explored for better separation of metabolites present in extracts. Using a common solvent system, toluene: ethyl acetate: formic acid (5 : 4:1, v/v/v), maximum separation of metabolites was achieved based on optimal bands with compactness in terms of resolution in TLC plates. Figures 1 and 2 show a TLC profiling of various extracts from eight different plant materials. The fluorescent image was analyzed under UV 254 and 366 nm using a TLC scanner III chamber. In the WE, EE, and HEE of *P. emblica*, a total number of 09, 13, and 12, respectively, metabolites were detected at 254 nm, while, at 366 nm, 10, 12, and 9 metabolites were recorded for the above-said extract, respectively. Under the wavelengths of 254 and 366 nm, the image of *P. nigrum* was studied, and a total number of 12, 10, 11 bands were detected in WE, EE, and HEE, respectively, at 254 nm, while, at 366 nm, the presence of 06, 09, and 06 bands was recorded for the said extracts of *P. nigrum*. The result of TLC fingerprinting of *T. cordifolia* showed 11, 11, and 14 metabolites for WE, EE, and HEE, respectively, at 254 nm, while for *T. cordifolia* at 366 nm, a total number of 05, 06, and 12 metabolites were detected in WE, EE, and HE, respectively. The TLC fingerprinting of *W. somnifera* detected 11 metabolites for each EE and HEE, while only 07 metabolites separated in WE at 254 nm, whereas WE, EE, and HEE of *W. somnifera* showed 03, 05, and 06 metabolites at 366 nm, respectively. A total of 11, 09, and 11 separated bands were detected in WE, EE, and HEE of *A. indica*, respectively, when observed under 254 nm, while 05, 05, and 09 metabolites were detected under 366 nm for the above-said extract, respectively. TLC fingerprint analysis of WE, EE, and HEE of *C. longa* showed 08, 06, and 08 prominent spots at 254 nm, respectively, while under 366 nm 05, 04, and 05 prominent spots were detected for the above-said extract, respectively. The TLC fingerprint analysis of *O. sanctum* extracts separated 11, 12, and 13 metabolites in WE, EE, and HEE, respectively. While scanning at 366 nm, ten metabolites were recorded for each extract of *O. sanctum*. The TLC fingerprint of *A. millefolium* extracts showed the presence of 03, 08, and 08 metabolites for WE, EE, and HEE, respectively, at 254 nm. At the same time, *A. millefolium* at 366 nm showed 02, 09, and 08 metabolites in WE, EE, and HEE, respectively. The plates scanned at 254 and 366 nm with respective areas at different *R*_f_ values and chromatogram of each extract are summarized in supportive information (Table S1 and Figures S1–S3).

### 3.4. Statistical Correlation to Determine the Metabolite Profiling of These Extracts

**Figure 3:**
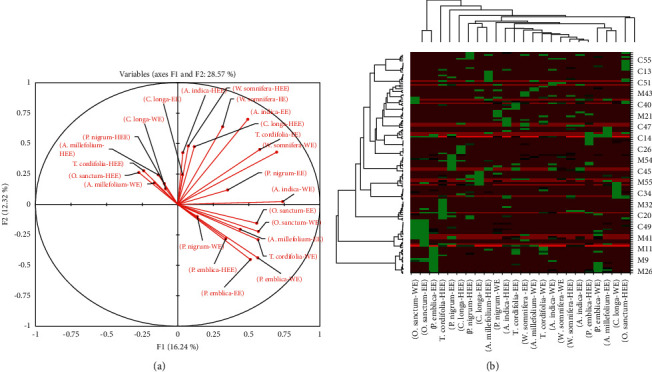
(a) Principal component analysis (PCA) score plots of variable factor map/correlation circles showing the different clusters of samples based on their metabolite abundance; (b) heatmap analysis based on metabolites present in different extracts of plant materials.

PCA was applied to identify metabolites of all three extracts of eight different Indian medicinal plants. The multidimensional dataset was reduced, and 119 (63 and 56) variables were categorized into two principal components (PCs), i.e., PC1 on the *X*‐axis and PC2 on the *Y*‐axis. The variables of the analyzed data were centered and transferred into unit variance. Using these two PCs, a total of 28.57% of the variance was enlightened, in which 16.24% was the first principal component (Dim.1/PC1) and 12.32% was the second principal component (Dim.2/PC2). Metabolites of HEE of *A. indica* are considered as a control closely clustered with another sample in the PCA score plot (Figure 3(a)). The maximum correlation was observed between metabolites of *O. sanctum*-EE and *O. sanctum*-WE (R 0.86), followed by *P. emblica*-EE and *P. emblica*-WE (R 0.84), *A. indica*-HEE and *W. somnifera*-HEE (R 0.67), *A. indica*-EE and *W. somnifera*-WE (R 0.64), *A. millefolium*-HEE and *A. millefolium*-WE (R 0.58), *W. somnifera*-WE and *T. cordifolia*-EE (R 0.55), *A. millefolium*-EE and *P. nigrum*-HEE (R 0.53), *A. indica*-WE and *W. somnifera*-WE (R 0.51), *W. somnifera*-EE and *W. somnifera*-WE (R 0.48), *T. cordifolia*-WE and *P. emblica*-EE (R 0.39), *A. millefolium*-WE and *O. sanctum*-HEE (R 0.36), *C. longa*-HEE and *C. longa*-WE (R 0.35), *C. longa*-EE and *P. nigrum*-WE (R 0.35), *O. sanctum*-WE and *A. indica*-WE (R 0.34), *T. cordifolia*-HEE and *P. nigrum*-HEE (R 0.34), *W. somnifera*-HEE and *W. somnifera*-EE (R 0.32), *P. emblica*-HEE and *P. emblica*-WE (R 0.25), *O. sanctum*-HEE and *A. indica*-HEE (R 0.20), *C. longa*-WE and *P. nigrum*-HEE (R 0.16), *T. cordifolia*-EE and *T. cordifolia*-WE (R 0.13), *P. nigrum*-HEE and *P. nigrum*-WE (R 0.08), *P. nigrum*-EE and *P. emblica*-EE (R 0.07), and *P. nigrum*-WE and *P. emblica*-WE (R 0.06) (details are shown in supportive information Table S2). The eigenvalues, percentage variance, and percentage cumulative details are in supportive information (Table S3). We can determine the major abundant metabolites that generated significant variations between the samples using metabolomic analysis. As a result, the metabolomic approach may be beneficial for standardizing plant materials based on metabolite patterns in various samples [16]. The discovery of pharmacological compounds from natural sources can benefit from the metabolomic analysis [12]. Similar metabolite patterns were found in *P. nigrum*-WE, *P. emblica*-HEE, *P. emblica*-EE, *P. emblica*-WE, *T. cordifolia*-WE, *A. millefolium*-EE, *O. sanctum*-WE, and *O. sanctum*-EE, according to the PCA score plot, whereas *A. indica*-WE, *P. nigrum*-EE, *W. somnifera*-WE, *T. cordifolia*-EE, *C longa*-HEE, *A. indica*-EE, *W. somnifera*-EE, *W. somnifera*-HEE, and *A. indica*-HEE were found in similar metabolite pattern in the PCA score plot. *C. longa*-EE, *C longa*-WE, *P. nigrum*-HEE, *A. millefolium*-HEE, *T. cordifolia*-HEE, *O. sanctum*-HEE, and *A. millefolium*-WE are in the same quadrant and having the same pattern of metabolites.

A heatmap was constructed based on the metabolites present in the sample using the MetaboAnalyst tool, which is used for unsupervised clustering. Metabolites were extracted using OPLS-DA. PCA was used to assess the general interrelationship between all samples of eight different plant materials in general. We provided a novel approach to analyzing the analytical data of major abundant metabolites. Heatmaps, on the other hand, are commonly employed for unsupervised clustering. Based on the abundance of their specific metabolites, the heatmaps revealed a significant separation among the samples. As a result, this approach might be utilized to identify metabolites that differentiate between eight different plant materials. The higher the abundance, the red the color, and the lesser the abundance, the green the color. The intensity of the color was proportional to the amount of metabolites present (Figure 3(b)).

### 3.5. Quantitative Estimation of Polyphenolic Compounds

Water extracts of the plant materials were chemically quantifying by polyphenolic markers. The linear regression calibration curves were plotted between peak area against the concentration and were linear for all standards, namely gallic acid (*r*^2^ = 0.99), quercetin (*r*^2^ = 0.98), and ferulic acid (*r*^2^ = 0.99) with good linear relationships. Well-separated gallic acid, quercetin, and ferulic acid bands were visualized at *R*_f_ 0.51 ± 0.03, 0.67 ± 0.03, and 0.75 ± 0.02, respectively. The percentage of gallic acid in *P. emblica* (4.75 ± 0.46%), quercetin in *C. longa* (4.39 ± 0.52%), and ferulic acid in *A. millefolium* (2.90 ± 0.37%) were found highest of total weight (w/w).

The linear regression calibration curves were plotted between peak area against the concentration for all standards, namely berberine (*r*^2^ = 0.99), piperine (*r*^2^ = 0.99), withaferin A (*r*^2^ = 0.99), and curcumin (*r*^2^ = 0.98) with good linear relationships. Well-separated bands of berberine, piperine, withaferin A, and curcumin were visualized at *R*_f_ 0.46, 0.69, 0.35, and 0.85, respectively. The percentage of piperine in *P. nigrum*, berberine in *T. cordifolia*, withaferin A in *W. somnifera*, and curcumin in *C. longa* were found to be 1.62 ± 0.01%, 1.04 ± 0.02%, 2.35 ± 0.04%, and 0.25 ± 0.05%, respectively, of total weight (w/w). The percentage of common polyphenolic compounds and some specific compounds present in water extracts of plant materials are shown in Table 4.

### 3.6. In vitro Immunomodulatory Activity

#### 3.6.1. Splenocyte Proliferation Assay

**Figure 4:**
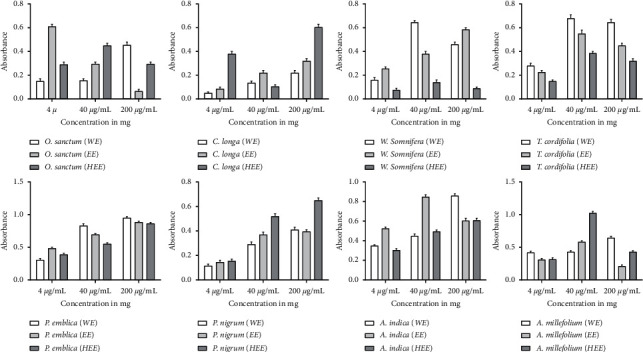
Splenocyte proliferation assay of different extracts of plant materials. (a) *O. sanctum*; (b) *C. longa*; (c) *T. cordifolia*; (d) *W. somnifera*; (e) *P. emblica*; (f) *P. nigrum*; (g) *A. indica*; (h) *A. millefolium.*

The immunomodulatory effect of the plant materials was investigated on isolated spleen cells. Three different concentrations of all plant extract were tested for splenocyte proliferation assay. The results in Figure 4 showed that *P. emblica* stimulates spleen cell proliferation in a dose-dependent manner in all three extracts. WE of *P. emblica* showed significantly higher proliferation, up to 97%, as compared to EE and HEE. A similar result showed all three doses of HEE of *P. nigrum* exhibited higher proliferation than other extracts. At 200 *μ*g/mL of HEE of *P. nigrum* showed a maximum of 65% spleen cell proliferation. WE of *T. cordifolia* stimulates the splenocyte proliferation, and a maximum of 71% growth was observed at 40 *μ*g/mL; after the increment of dose 200 *μ*g/mL, the results showed no significant increase in the splenocyte proliferation. WE of *W. somnifera* showed a maximum of 65% splenocyte proliferation at 40 *μ*g/mL, and it was significantly higher than other extracts. WE and EE of *C. longa* showed splenocyte proliferation in a dose-dependent manner, but HEE stimulates maximum growth at 200 *μ*g/mL. WE of *A. indica* also stimulates splenocyte proliferation in a dose-dependent manner, and maximum growth was observed at 200 *μ*g/mL. All three extracts of *O. sanctum* and *A. millefolium* stimulate splenocyte proliferation, but they did not in a dose-dependent manner. IMMU-21 is an Ayurvedic polyherbal formulation containing extracts of *P. emblica*, *O. sanctum*, *W. somnifera,* and *T. cordifolia* reported to have splenocyte proliferation activity [17]. The effect of different extracts of plant material on splenocyte proliferation has been shown in Figure 4.

#### 3.6.2. Pinocytic Activity Assay

**Figure 5:**
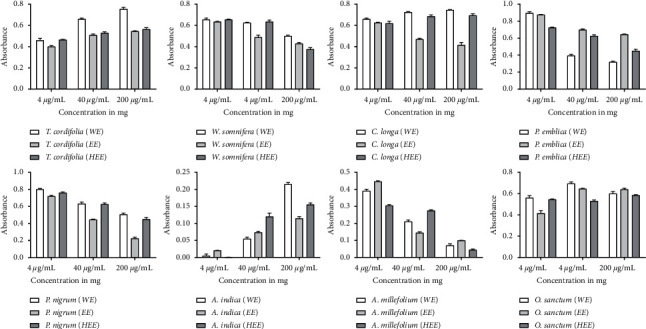
Pinocytic activity of different extracts of plant materials. (a) *T. cordifolia;* (b) *W. somnifera;* (c) *C. longa;* (d) *P. emblica;* (e) *P. nigrum;* (f) *A. indica;* (g) *A. millefolium;* (h) *O. sanctum.*

To demonstrate the immunomodulatory effects of these plant materials, we used neutral red which is readily uptaken by macrophages. The enhancement of pinocytic activity was expressed as an increment in neutral red concentration in cells treated with external stimuli compared to untreated cells. The results in Figure 5 showed that the extracts' enhancement of the activity was examined at different concentrations (4 *μ*g/mL, 40 *μ*g/mL, and 200 *μ*g/mL). WE of *T. cordifolia* exhibited a dose-dependent pinocytic activity, and a maximum of 77% activity was observed at the highest test dose, 200 *μ*g/mL. At the same time, its EE and HEE showed almost steady pinocytic activity, but it was less than the activity exhibited in water extract. Literature also supports the dose-dependent pinocytic activity of the water extract of *T. cordifolia* [18]. WE of *P. emblica* showed a maximum of 91% pinocytic activity at 4 *μ*g/mL, and the activity was decreased upon concentration increment. Similarly, WE of *P. nigrum* showed maximum pinocytic activity at 4 *μ*g/mL, and the activity decreased upon the dose increment in all three extracts. WE and HEE of *C. longa* showed almost steady pinocytic activity of all three doses. *W. somnifera* did not show any significant differences among the three different extracts. All three extracts of *A. millefolium* showed a significant reduction in pinocytic activity upon dose increment. WE of *O. sanctum* at 40 *μ*g/mL showed the highest pinocytic activity, but after the dose increment, the activity was decreased.

## 4. Discussion

Traditional medicinal plants have a long history and are still used as primary healthcare options, particularly among indigenous peoples. Traditional medicinal plants are becoming increasingly popular as medical alternatives in developed and developing countries to treat various disorders, including immune disease [19].

Several medicinal plants have been used for immunological problems in Ayurveda and Unani, either individually or in polyherbal formulations [2, 3]. The fruits of *P. emblica* and *P. nigrum*, stem of *T. cordifolia*, rhizome of *C. longa,* leaves of *O. sanctum* and *A. millefolium,* roots of *W. somnifera,* and stem bark of *A. indica* were screened for the studies based on the literature. These plant materials were extracted in three different solvents (water, ethanol, and hydroethanol). We obtained a higher yield of WE as compared to others in the conventional extraction process. However, the HEE extractive values of some plants were higher than WE and EE extractive values.

Phenolic and flavonoids are well-known antioxidants that have long been attention due to their health-promoting, disease-curing, and disease prevention properties. Many phenolic and flavonoid compounds have been reported to have antioxidants and immune-boosting properties [20]. The antioxidant capacity of all three extracts of each plant material was expressed as equivalent of ascorbic acid. Table 3 shows the DPPH radical scavenging potential and reducing power capacity of the plant extracts. Most of the WE of plant materials shows strong antioxidant potential due to the contents of the secondary metabolites. The extraction of phenolics from plant material with strong antioxidant activity is affected by a number of factors. Among them, extraction method and choice of solvent are the most significant. A mixture of water organic solvents is the most suitable for extraction of TPC from plant material [21]. Hydroxyl group present in the flavonoids mediate their antioxidant activity by scavenging free radicals and by chelating metal ions. Flavonoids contain a wide range of chemical and biological activities including antioxidant and free radical scavenging properties. Flavonoids have increased interest as they show beneficial health properties due to their potential antioxidant, anti-inflammatory, and anticancer activities.

One of the major problems for herbal-based products is their quality control analysis. TLC profiling is commonly used to obtain metabolite patterns from any extract of the plant materials. If any plant materials or their extract has the same TLC pattern, it must have the same biological activity. The developed method was reproducible, and their separated metabolite pattern is shown in Figures 1 and 2 at 254 and 366 nm, respectively. The TLC profiling of the plant materials can be used for quality control and regulatory bodies to ensure the product quality and safety [22].

In PCA, two principal components might explain a total of 28.57% of the variance. There was a significant variance in the precise clustering of metabolites detected in extracts of plant materials at varying *R*_f_ value. The PCA score plot shows that some extract contained comparable types of metabolites. The 119 (63 and 56 metabolites) variables in the multidimensional datasets were transformed into two principal components, which were designated as *x*-axis (PCI) and *y*-axis (PC2) in the study. The variables were centered and scaled to unit variance in the data that was studied. The first principal component (PC1) accounted for 16.24% of the variation in the data, whereas the second principal component (PC2) accounted for 12.32%. PCA score plot also suggests the correlation of the extracts does not depend on their type of the extract. The heatmap, which is often used for unsupervised clustering, was generated using OPLS-DA analysis to identify possible candidates of importance. The difference between extracts of plant materials was separated significantly using heatmap analysis [12]. With the metabolomics platform, we were able to develop a heatmap visualization that could be used in computational systems approach to determine the similarity and dissimilarity of metabolites in the samples. Only metabolites with a higher proportion of contribution were shown in the heatmap among 119 metabolites in extracts.

Immunopharmacology is a comparatively new and emerging division of pharmacology that goals to discover new immunomodulators. Plant and its extracts are extensively explored in different parts of the world for their promising immunomodulatory properties. The immunomodulatory effect of the plant materials was investigated on spleen cells and peritoneal macrophages, isolated from mice. The results prove that *P. emblica* stimulates spleen cell proliferation in a dose-dependent manner in all three extracts but WE showed significantly higher cell proliferation up to 97% as compared to other extract. *P. emblica* is an essential medicinal plant in AYUSH system of medicine specially in Ayurveda and Unani and is also used as a dietary supplement. It possesses various properties such as anticancer and immune deficiency [23]. *P. emblica* has significant immunostimulatory effects on cellular immune response [24]. The result of WE of *P. emblica*, *T. cordifolia*, *W. somnifera*, and *A. indica* showed highest proliferation. The root extract of *W. somnifera* significantly enhances splenocyte proliferation [25]. This result suggests that the metabolites present in WE may stimulate T lymphocyte proliferation, supposedly inhibiting the cellular immune response [26]. Though HEE of *P. nigrum* stimulates spleen cell proliferation in a dose-dependent manner, the literature also supports its spleen cell proliferation [27]. *O. sanctum* and *A. millefolium* extracts also stimulate spleen cell proliferation; these results supported the previous finding [28].

The immunomodulator effect of the plant extract also investigated on thioglycolate-elicited peritoneal macrophages from mice. Because of their high adhesion capacity, peritoneal macrophages can be enhanced from peritoneal lavage fluid. As a result, the macrophage population is significantly isolated from the other cells in the peritoneal cavity (B cells, dendritic cells, neutrophils, T cells, NK cells, etc.) [29]. Only WE of *T. cordifolia* exhibited a dose-dependent pinocytic activity and modulated the immune system; its water extract is superior to alcoholic extract [30]; however WE of *P. emblica* and *P. nigrum* showed maximum pinocytic activity at lowest dose. *P. nigrum* showed immunomodulatory potential through macrophage proliferation as well as splenocyte proliferation [31]. *W. somnifera* did not show any significant differences among three different extracts. It stimulates the phagocytosis of macrophages, as seen from the increased pigmented macrophages [32]. WE and HEE of *C. longa* showed the dose-dependent pinocytic activity but did not show any significant difference.

## 5. Conclusion

In this study, some important plant materials used in AYUSH were explored for their antioxidant and immunomodulatory activity. Selected plant materials were characterized through their metabolic content, quantification of some specific and common markers. In spite of having low phenolic and flavonoid contents in water extract as compared to other extract, it showed better immunomodulatory and antioxidant activity. Through PCA, it also revealed different extracts of one plant materials having either similar metabolite pattern or different metabolite patterns. From the above study, the traditional use of water extract in AYUSH system may be justifiable. Further, use of water extract in Indian population since long may warrant for its safety, whereas other extracts may need toxicity profiling before they explore in human subjects. This study provides a confidential support to the AYUSH system of medicine in which water extract is recommended as major in their formulations. The developed TLC method can be used for quality control analysis of plant materials having similar metabolic profile.

## Figures and Tables

**Table 1:** Percentage yield of different extracts of plant materials.

S. no.	Plant sample	Percentage yield (mean ± SD)		
		WE	EE	HEE
1	*Phyllanthus emblica*	51.42 ± 2.51	44.81 ± 3.45^ns^	41.51 ± 2.54^*∗*^
2	*Piper nigrum*	9.60 ± 1.08	8.21 ± 1.24^ns^	10.83 ± 1.56^ns^
3	*Withania somnifera*	16.44 ± 1.28	11.22 ± 0.47^*∗∗*^	13.62 ± 1.25^*∗*^
4	*Tinospora cordifolia*	12.61 ± 1.09	4.54 ± 0.48^*∗∗∗*^	7.42 ± 0.08^*∗∗∗*^
5	*Curcuma longa*	13.84 ± 1.04	9.84 ± 1.11^*∗*^	11.04 ± 2.12^ns^
6	*Ocimum sanctum*	8.44 ± 0.87	11.81 ± 0.65^*∗∗*^	15.63 ± 1.07^*∗∗∗*^
7	*Azadirachta indica*	20.22 ± 1.54	8.12 ± 0.07^*∗∗∗*^	14.04 ± 0.12^*∗∗∗*^
8	*Achillea millefolium*	10.41 ± 0.89	7.23 ± 0.21^*∗∗*^	12.51 ± 0.47^*∗*^

Data are expressed as mean ± SD (*n* = 3). One-way ANOVA followed by Tukey's multiple comparisons test. Compared to WE (water extract): ^*∗*^*p* < 0.05, ^*∗∗*^*p* < 0.01, ^*∗∗∗*^*p* < 0.001; ns *p*>0.05. ^*∗*^WE = water extract; EE = ethanolic extract; HEE = hydroethanolic extract.

**Table 2:** Total phenolic and flavonoid contents of different extracts of plant materials.

S. no.	Plant sample	TPC (%) Mean ± SD			TFC (%) Mean ± SD		
		WE	EE	HEE	WE	EE	HEE
1	*Phyllanthus emblica*	31.25 ± 0.94	27.55 ± 0.54^*∗*^	42.35 ± 1.70^*∗∗∗*^	16.24 ± 1.91	10.21 ± 1.09^*∗∗*^	20.15 ± 1.22^*∗*^
2	*Piper nigrum*	12.31 ± 1.21	10.54 ± 0.90^ns^	20.38 ± 2.16^*∗∗*^	06.44 ± 0.79	05.24 ± 0.84^ns^	11.56 ± 1.49^*∗∗*^
3	*Withania somnifera*	26.25 ± 0.45	22.75 ± 1.02^ns^	38.42 ± 1.54^*∗∗∗*^	11.24 ± 1.15	09.92 ± 0.99^ns^	19.58 ± 1.58^*∗∗∗*^
4	*Tinospora cordifolia*	14.11 ± 1.10	11.85 ± 0.94^ns^	19.30 ± 1.24^*∗∗*^	06.55 ± 0.62	04.18 ± 0.83^ns^	14.85 ± 1.88^*∗∗∗*^
5	*Curcuma longa*	09.91 ± 0.17	8.12 ± 0.90^ns^	12.78 ± 1.10^*∗*^	05.87 ± 1.14	04.09 ± 0.80^ns^	13.77 ± 1.30^*∗∗∗*^
6	*Ocimum sanctum*	17.21 ± 1.26	13.89 ± 0.95^*∗*^	26.48 ± 1.55^*∗∗∗*^	10.45 ± 1.67	07.35 ± 0.77^ns^	18.08 ± 2.62^*∗∗*^
7	*Azadirachta indica*	25.54 ± 1.00	19.57 ± 0.68^*∗*^	34.46 ± 2.99^*∗∗*^	14.45 ± 1.54	12.34 ± 1.91^ns^	17.64 ± 1.15^ns^
8	*Achillea millefolium*	14.21 ± 1.05	13.54 ± 1.15^ns^	19.51 ± 2.18^*∗*^	06.87 ± 0.86	04.28 ± 0.80^*∗*^	10.24 ± 0.81^*∗∗*^

Data are expressed as mean ± SD (*n* = 3). One-way ANOVA followed by Tukey's multiple comparison tests. Compared to WE (water extract): ^*∗*^*p* < 0.05, ^*∗∗*^*p* < 0.01, ^*∗∗∗*^*p* < 0.001; ns *p*>0.05. ^*∗*^TPC = total phenolic content; TFC = total flavonoid content; WE = water extract; EE = ethanolic extract; HEE = hydroethanolic extract.

**Table 3:** DPPH free radical scavenging and reducing power capacity of different extracts of plant materials.

S. no.	Plant sample	DPPH scavenging activity (IC_50_ (*μ*g/mL))			Reducing power capacity (*μ*g/mL) Mean ± SD		
		WE	EE	HEE	WE	EE	HEE
1	*Phyllanthus emblica*	97.26 ± 1.14	124.89 ± 3.45^*∗∗∗*^	115.45 ± 2.45^*∗∗∗*^	100 ± 10	160 ± 20^*∗∗*^	120 ± 10^ns^
2	*Piper nigrum*	112.18 ± 2.11	222.34 ± 2.36^*∗∗∗*^	210.89 ± 0.45^*∗∗∗*^	140 ± 20	240 ± 10^*∗∗∗*^	220 ± 10^*∗∗*^
3	*Withania somnifera*	85.96 ± 3.42	115.65 ± 3.63^*∗∗∗*^	91.26 ± 1.11^ns^	100 ± 10	140 ± 20^*∗*^	120 ± 10^ns^
4	*Tinospora cordifolia*	105.65 ± 3.33	185.56 ± 2.56^*∗∗∗*^	177.23 ± 2.31^*∗∗∗*^	120 ± 10	200 ± 10^*∗∗*^	180 ± 20^*∗∗*^
5	*Curcuma longa*	199.15 ± 0.42	285.47 ± 4.56^*∗∗∗*^	205.66 ± 1.75^ns^	200 ± 30	300 ± 10^*∗∗*^	220 ± 20^ns^
6	*Ocimum sanctum*	201.56 ± 1.11	245.25 ± 3.21^*∗∗∗*^	223.45 ± 3.33^*∗∗∗*^	240 ± 20	280 ± 20^ns^	220 ± 10^ns^
7	*Azadirachta indica*	156.83 ± 0.88	171.22 ± 2.56^*∗∗∗*^	162.32 ± 2.11^*∗*^	160 ± 10	200 ± 20^ns^	180 ± 30^ns^
8	*Achillea millefolium*	185.65 ± 1.23	145.56 ± 2.22^*∗∗∗*^	152.23 ± 2.56^*∗∗∗*^	160 ± 20	180 ± 20^ns^	200 ± 20^ns^
9	Ascorbic acid	55.37 ± 1.25	80 ± 10				

Data are expressed as mean ± SD (*n* = 3). One-way ANOVA followed by Tukey's multiple comparisons test. Compared to WE (water extract): ^*∗*^*p* < 0.05, ^*∗∗*^*p* < 0.01, ^*∗∗∗*^*p* < 0.001; ns *p*>0.05.^*∗*^WE = water extract; EE = ethanolic extract; HEE = hydroethanolic extract.

**Table 4:** Percentage of common metabolites (gallic acid, quercetin, and ferulic acid) and some specific metabolites present in water extracts of different plant materials.

	*P. emblica*	*P. nigrum*	*T. cordifolia*	*W. somnifera*	*A. indica*	*C. longa*	*O. sanctum*	*A. millefolium*
Gallic acid	4.75 ± 0.46	0.26 ± 0.08	0.65 ± 0.11	0.27 ± 0.08	0.40 ± 0.16	—	—	0.16 ± 0.08
Quercetin	0.54 ± 0.09	1.68 ± 0.29	0.77 ± 0.15	0.41 ± 0.10	0.95 ± 0.13	4.39 ± 0.52	2.01 ± 0.28	—
Ferulic acid	1.08 ± 0.12	1.12 ± 0.22	0.31 ± 0.07	0.38 ± 0.11	0.30 ± 0.06	1.71 ± 0.31	1.13 ± 0.17	2.90 ± 0.37
Berberine	—	—	1.04 ± 0.02	—	—	—	—	—
Piperine	—	1.62 ± 0.01	—	—	—	—	—	—
Withaferin A	—	—	—	2.35 ± 0.04	—	—	—	—
Curcumin	—	——	—	—	—	0.25 ± 0.05	—	—

## Data Availability

The data used to support the findings of this study are available from the corresponding author upon request.

## Supplementary Materials

Supplementary Materials Supplementary Table S1 A: TLC profile of all three extracts of different plant materials scanned at 254 nm. Supplementary Table S1 B: TLC profile of all three extracts of different plant materials scanned at 366 nm. Supplementary Table S2: correlation matrix (Pearson n) of variables. Supplementary Table S3: eigenvalues of variables from principal component analysis (PCA). Figure S1: developed thin-layer chromatogram of water extract (WE) of *P. emblica* (A1), *P. nigrum* (B1), *T. cordifolia* (C1), *W. somnifera* (D1), *A. indica* (E1), *C. longa* (F1), *O. sanctum* (G1), and *A. millefolium* (H1) at 254 nm and *P. emblica* (A2), *P. nigrum* (B2), *T. cordifolia* (C2), *W. somnifera* (D2), *A. indica* (E2), *C. longa* (F2), *O. sanctum* (G2), and *A. millefolium* (H2) at 366 nm. Figure S2: developed thin-layer chromatogram of ethanolic extract (EE) of *P. emblica* (A1), *P. nigrum* (B1), *T. cordifolia* (C1), *W. somnifera* (D1), *A. indica* (E1), *C. longa* (F1), *O. sanctum* (G1), and *A. millefolium* (H1) at 254 nm and *P. emblica* (A2), *P. nigrum* (B2), *T. cordifolia* (C2), *W. somnifera* (D2), *A. indica* (E2), *C. longa* (F2), *O. sanctum* (G2), and *A. millefolium* (H2) at 366 nm. Figure S3: developed thin-layer chromatogram of hydroethanolic extract (HEE) of *P. emblica* (A1), *P. nigrum* (B1), *T. cordifolia* (C1), *W. somnifera* (D1), *A. indica* (E1), *C. longa* (F1), *O. sanctum* (G1), and *A. millefolium* (H1) at 254 nm and *P. emblica* (A2), *P. nigrum* (B2), *T. cordifolia* (C2), *W. somnifera* (D2), *A. indica* (E2), *C. longa* (F2), *O. sanctum* (G2), and *A. millefolium* (H2) at 366 nm.

## References

[1] WHO (2019). *WHO Global Report on Traditional and Complementary Medicine*.

[2] Ahmad S., Zahiruddin S., Parveen B. (2021). Indian medicinal plants and formulations and their potential against COVID-19-preclinical and clinical research. *Frontiers in Pharmacology*.

[3] Kumar D., Arya V., Kaur R., Bhat Z. A., Gupta V. K., Kumar V. (2012). A review of immunomodulators in the Indian traditional health care system. *Journal of Microbiology, Immunology, and Infection*.

[4] (2000). *General Guidelines for Methodologies on Research and Evaluation of Traditional Medicine World Health Organization*.

[5] Liang Y. Z., Xie P., Chan K. (2004). Quality control of herbal medicines. *Journal of chromatography. B, Analytical technologies in the biomedical and life sciences*.

[6] Ong E. S. (2002). Chemical assay of glycyrrhizin in medicinal plants by pressurized liquid extraction (PLE) with capillary zone electrophoresis (CZE). *Journal of Separation Science*.

[7] Chothani D. L., Patel M. B., Mishra S. H. (2012). HPTLC fingerprint profile and isolation of marker compound of ruellia tuberosa. *Chromatography Research International*.

[8] Hajimehdipoor H., Khanavi M., Zahedi H. (2009). Fingerprint study of thymus spp. by TLC. *Journal of Medicinal Plants*.

[9] Nile S. H., Park S. W. (2014). HPTLC analysis, antioxidant and antigout activity of indian plants. *Iranian Journal of Pharmaceutical Research: IJPR*.

[10] IP (2007). *Indian Pharmacopoeia*.

[11] API (2008). *The Ayurvedic Pharmacopoeia of India*.

[12] Khan W., Parveen R., Chester K., Parveen S., Ahmad S. (2017). Hypoglycemic potential of aqueous extract of Moringa oleifera leaf and in vivo GC-MS metabolomics. *Frontiers in Pharmacology*.

[13] Zekič J., Vovk I., Glavnik V. (2021). Extraction and analyses of flavonoids and phenolic acids from canadian goldenrod and giant goldenrod. *Forests*.

[14] Khan W., Chester K., Anjum V. (2017). Chromatographic profiling of Pancharishta at different stages of its development using HPTLC, HPLC, GC-MS and UPLC-MS. *Phytochemistry Letters*.

[15] Shi L., Fu Y. (2011). Isolation, purification, and immunomodulatory activity in vitro of three polysaccharides from roots of Cudrania tricuspidata. *Acta Biochimica et Biophysica Sinica*.

[16] Lee K.-M., Jeon J.-Y., Lee B.-J., Lee H., Choi H.-K. (2017). Application of metabolomics to quality control of natural product derived medicines. *Biomolecules & Therapeutics*.

[17] Nemmani K. V., Jena G. B., Dey C. S., Kaul C. L., Ramarao P. (2002). Cell proliferation and natural killer cell activity by polyherbal formulation, Immu-21 in mice. *Indian Journal of Experimental Biology*.

[18] More P., Pai K. (2017). Effect of tinospora cordifolia (Guduchi) on the phagocytic and pinocytic activity of murine macrophages in vitro. *Indian Journal of Experimental Biology*.

[19] Wachtel-Galor S., Benzie I. F. F. (2011). Herbal medicine: an introduction to its history, usage, regulation, current trends, and research needs. *Herbal Medicine: Biomolecular and Clinical Aspects*.

[20] Tungmunnithum D., Thongboonyou A., Pholboon A., Yangsabai A. (2018). Flavonoids and other phenolic compounds from medicinal plants for pharmaceutical and medical aspects: an overview. *Medicine*.

[21] Venkatesan T., Choi Y.-W., Kim Y.-K. (2019). Impact of different extraction solvents on phenolic content and antioxidant potential of pinus densiflora bark extract. *BioMed Research International*.

[22] Parveen R., Zahiruddin S., Charegaonkar A., Khale A., Mallick S. (2020). Chromatographic profiling of rose petals in unani formulations (Gulkand, Arq-e-gulab, and rose sharbat) using hptlc and GC-MS. *Journal of AOAC International*.

[23] (2011). Antioxidant, mmunomodulatory and anticancer activities of Emblica officinalis: an overview. *International Research Journal of Pharmacy*.

[24] Phetkate P., Kummalue T., U-Pratya Y., Kietinun S. (2012). Significant increase in cytotoxic T lymphocytes and natural killer cells by triphala: a clinical phase i study. *Evidence-based Complementary and Alternative Medicine*.

[25] Malik F., Singh J., Khajuria A. (2007). A standardized root extract of Withania somnifera and its major constituent withanolide-A elicit humoral and cell-mediated immune responses by up regulation of Th1-dominant polarization in BALB/c mice. *Life Sciences*.

[26] Cano L. E., Lopera D. E. (2013). Introduction to T and B lymphocytes. *Autoimmunity from bench to bedside*.

[27] Haq I. U., Imran M., Nadeem M., Tufail T., Gondal T. A., Mubarak M. S. (2021). Piperine: a review of its biological effects. *Phytotherapy Research*.

[28] Goel A., Singh D., Bhatia A. (2010). Effect of Ocimum sanctum extract on the induction of IFN-*γ* and IL-10 cytokines and their m-RNA expression. *Journal of Immunology and Immunopathology*.

[29] Ghosn E. E. B., Cassado A. A., Govoni G. R. (2010). Two physically, functionally, and developmentally distinct peritoneal macrophage subsets. *Proceedings of the National Academy of Sciences*.

[30] Manjrekar P. N., Jolly C. I., Narayanan S. (2000). Comparative studies of the immunomodulatory activity of Tinospora cordifolia and Tinospora sinensis. *Fitoterapia*.

[31] Saravanan P., Mohamed M. S. N., Jaikumar K., Anand D. (2017). Assessment of cytotoxic and immunomodulatory properties of piper nigrum linn. (White pepper) seed extract. *International Journal of Pharmaceutical Sciences and Drug Research*.

[32] Davis L., Kuttan G. (2000). Immunomodulatory activity of Withania somnifera. *Journal of Ethnopharmacology*.

