# Supplementary material for: TLC-Based Metabolite Profiling and Bioactivity-Based Scientific Validation for Use of Water Extracts in AYUSH Formulations
Source: Evid Based Complement Alternat Med. 2021 Dec 31;2021:2847440. doi: 10.1155/2021/2847440 (PMC8741349; doi:10.1155/2021/2847440)
Supplement: Supplementary Materials — Supplementary Table S1 A: TLC profile of all three extracts of different plant materials scanned at 254 nm. Supplementary Table S1 B: TLC profile of all three extracts of different plant materials scanned at 366 nm. Supplementary Table S2: correlation matrix (Pearson n) of variables. Supplementary Table S3: eigenvalues of variables from principal component analysis (PCA). Figure S1: developed thin-layer chromatogram of water extract (WE) of P. emblica (A1), P. nigrum (B1), T. cordifolia (C1), W. somnifera (D1), A. indica (E1), C. longa (F1), O. sanctum (G1), and A. millefolium (H1) at 254 nm and P. emblica (A2), P. nigrum (B2), T. cordifolia (C2), W. somnifera (D2), A. indica (E2), C. longa (F2), O. sanctum (G2), and A. millefolium (H2) at 366 nm. Figure S2: developed thin-layer chromatogram of ethanolic extract (EE) of P. emblica (A1), P. nigrum (B1), T. cordifolia (C1), W. somnifera (D1), A. indica (E1), C. longa (F1), O. sanctum (G1), and A. millefolium (H1) at 254 nm and P. emblica (A2), P. nigrum (B2), T. cordifolia (C2), W. somnifera (D2), A. indica (E2), C. longa (F2), O. sanctum (G2), and A. millefolium (H2) at 366 nm. Figure S3: developed thin-layer chromatogram of hydroethanolic extract (HEE) of P. emblica (A1), P. nigrum (B1), T. cordifolia (C1), W. somnifera (D1), A. indica (E1), C. longa (F1), O. sanctum (G1), and A. millefolium (H1) at 254 nm and P. emblica (A2), P. nigrum (B2), T. cordifolia (C2), W. somnifera (D2), A. indica (E2), C. longa (F2), O. sanctum (G2), and A. millefolium (H2) at 366 nm. [file 2847440.f1.zip › 2847440.f1/Figure S3 (1).pdf]

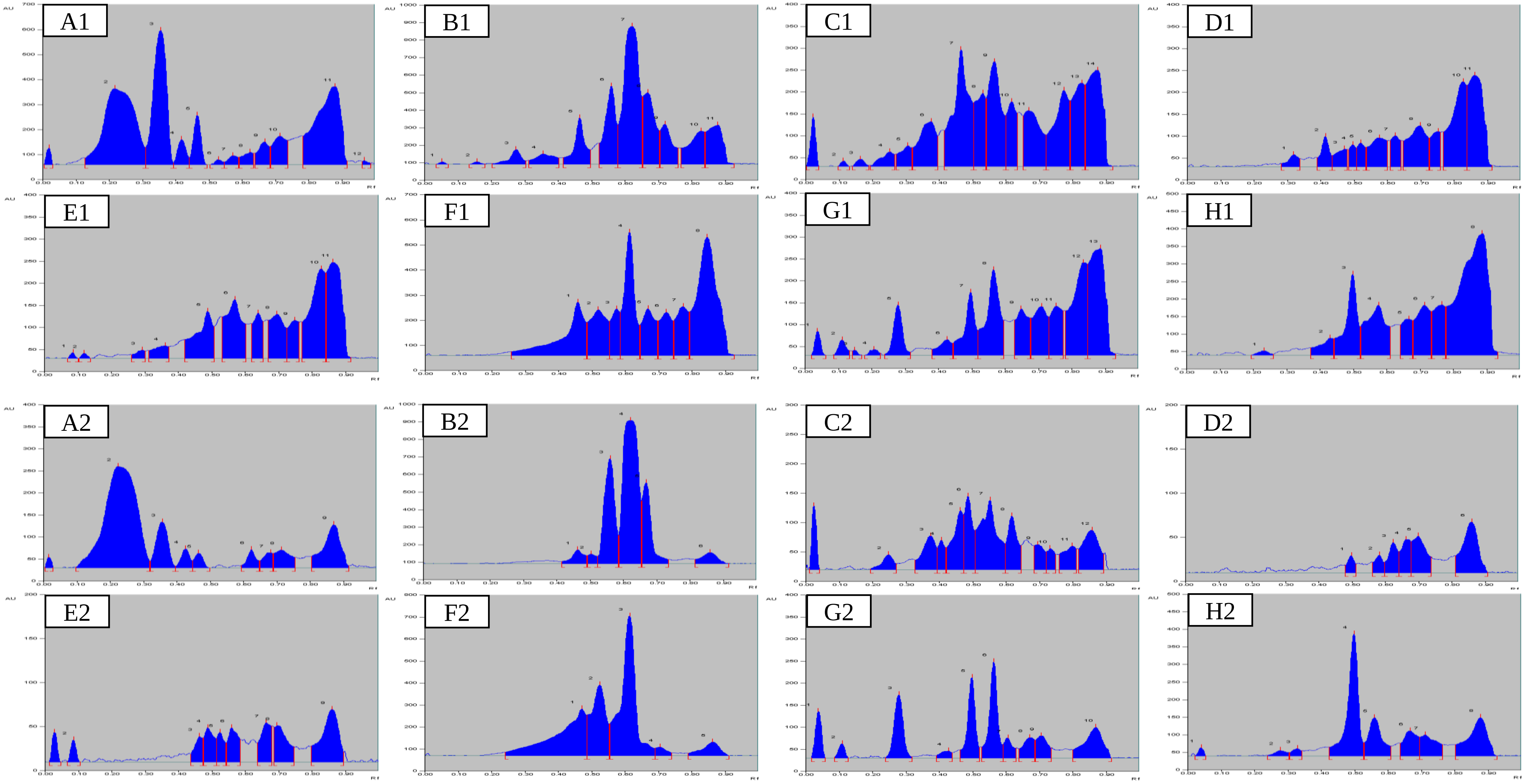

Figure S3: Developed thin layer chromatogram of Hydroethanolic Extracts (HEE) of *P. emblica* (A1), *P. nigrum* (B1), *T. cordifolia* (C1), *W. somnifera* (D1), *A. indica* (E1), *C. longa* (F1), *O. sanctum* (G1), *A. millefolium* (H1) at 254 nm and *P. emblica* (A2), *P. nigrum* (B2), *T. cordifolia* (C2), *W. somnifera* (D2), *A. indica* (E2), *C. longa* (F2), *O. sanctum* (G2), *A. millefolium* (H2) at 366 nm.
