# Supplementary material for: TLC-Based Metabolite Profiling and Bioactivity-Based Scientific Validation for Use of Water Extracts in AYUSH Formulations
Source: Evid Based Complement Alternat Med. 2021 Dec 31;2021:2847440. doi: 10.1155/2021/2847440 (PMC8741349; doi:10.1155/2021/2847440)
Supplement: Supplementary Materials — Supplementary Table S1 A: TLC profile of all three extracts of different plant materials scanned at 254 nm. Supplementary Table S1 B: TLC profile of all three extracts of different plant materials scanned at 366 nm. Supplementary Table S2: correlation matrix (Pearson n) of variables. Supplementary Table S3: eigenvalues of variables from principal component analysis (PCA). Figure S1: developed thin-layer chromatogram of water extract (WE) of P. emblica (A1), P. nigrum (B1), T. cordifolia (C1), W. somnifera (D1), A. indica (E1), C. longa (F1), O. sanctum (G1), and A. millefolium (H1) at 254 nm and P. emblica (A2), P. nigrum (B2), T. cordifolia (C2), W. somnifera (D2), A. indica (E2), C. longa (F2), O. sanctum (G2), and A. millefolium (H2) at 366 nm. Figure S2: developed thin-layer chromatogram of ethanolic extract (EE) of P. emblica (A1), P. nigrum (B1), T. cordifolia (C1), W. somnifera (D1), A. indica (E1), C. longa (F1), O. sanctum (G1), and A. millefolium (H1) at 254 nm and P. emblica (A2), P. nigrum (B2), T. cordifolia (C2), W. somnifera (D2), A. indica (E2), C. longa (F2), O. sanctum (G2), and A. millefolium (H2) at 366 nm. Figure S3: developed thin-layer chromatogram of hydroethanolic extract (HEE) of P. emblica (A1), P. nigrum (B1), T. cordifolia (C1), W. somnifera (D1), A. indica (E1), C. longa (F1), O. sanctum (G1), and A. millefolium (H1) at 254 nm and P. emblica (A2), P. nigrum (B2), T. cordifolia (C2), W. somnifera (D2), A. indica (E2), C. longa (F2), O. sanctum (G2), and A. millefolium (H2) at 366 nm. [file 2847440.f1.zip › 2847440.f1/Supportive information.pdf]

SUPPORTIVE INFORMATIONS

Table S1 A: TLC profile of all three extract of different plant materials scanned at 254 nm.

Plant		*P. emblica*			*P. nigrum*			*T. cordifolia*			*W. somnifera*			*A. indica*			*C. longa*			*O. sanctum*			*A. millefolium*		
Extract		WE	EE	HEE	WE	EE	HEE	WE	EE	HEE	WE	EE	HEE	WE	EE	HEE	WE	EE	HEE	WE	EE	HEE	WE	EE	HEE
Metabolites	Rf																								
M1	0.02	-	567.1	813.1	-	459.5	-	394.2	-	1555	-	-	-	239.5	-	-	-	-	-	-	785.1	-	-	-	-
M2	0.04	-	-	-	159	-	-	-	348	-	286	225	-	539	249	-	110	-	-	965	-	831	-	-	-
M3	0.05	-	1862	-	-	-	203.8	1085	1152	-	-	-	-	-	-	-	-	-	-	-	-	-	-	-	-
M4	0.08	1277	1491	-	-	-	-	-	362	-	-	-	-	-	-	-	-	-	-	-	-	-	-	-	-
M5	0.1	-	336	-	276.6	-	-	-	-	-	-	-	-	-	-	160.6	-	-	-	1943	2307	-	-	533.3	-
M6	0.11	-	-	-	-	-	-	-	-	183	-	-	-	481	-	178	-	-	-	-	-	735	-	-	-
M7	0.13	-	-	-	-	-	-	-	-	-	-	-	-	-	-	-	-	-	-	-	177	-	-	-	-
M8	0.16	-	-	-	-	-	264	-	-	376	-	-	-	-	-	-	-	-	-	-	-	157	-	-	-
M9	0.19	-	1935	-	-	-	-	-	-	-	-	-	-	402.8	-	-	-	-	-	322.7	392.3	-	-	-	-

M22	M21	M20	M19	M18	M17	M16	M15	M14	M13	M12	M11	M10
0.42	0.4	0.39	0.37	0.36	0.35	0.34	0.32	0.29	0.27	0.24	0.23	0.22
-	-	8004.5	5879	-	-	-	-	-	417.4	-	-	-
-	-	20919	-	-	-	-	-	-	-	-	1500	-
2304	-	-	-	-	19508	-	-	-	-	-	-	26603
-	2602	-	-	-	-	-	-	-	-	679.7	-	-
4604	-	-	-	-	-	-	-	-	-	416.5	-	-
-	-	-	-	-	3116.2	-	-	-	2668	-	-	-
-	1019	-	-	791	-	-	-	-	-	-	452	-
1526	-	-	-	-	-	1094	-	-	-	-	354.1	-
-	-	-	4767	-	-	-	1494	-	-	1185	-	-
-	-	-	-	-	-	-	-	-	-	-	-	-
-	-	-	-	-	818.4	-	-	-	-	-	-	-
-	1544	-	-	-	-	-	800	-	-	-	-	-
1439	-	-	-	-	-	-	-	-	-	1706	-	-
-	-	-	-	-	-	-	546	-	-	719.5	-	-
-	-	-	-	1212	-	-	-	490	-	-	-	-
-	-	-	-	-	-	-	-	-	-	-	-	340
-	-	-	-	-	-	-	-	-	-	-	-	-
-	-	-	-	-	-	-	-	-	-	-	-	-
-	-	-	496.8	-	-	-	-	-	-	-	-	-
-	-	-	-	-	-	-	-	-	-	-	-	-
-	-	-	-	-	-	-	-	-	2785	-	-	310.5
-	-	-	-	-	-	-	-	-	-	-	-	-
-	-	1335.1	-	-	-	-	-	626	-	-	-	-
-	-	-	-	-	-	-	-	-	-	-	376.3	-

M34	M33	M32	M31	M30	M29	M28	M27	M26	M25	M24	M23
0.56	0.55	0.54	0.53	0.52	0.51	0.5	0.49	0.48	0.47	0.44	0.43
-	-	-	-	-	-	-	-	20908	-	-	-
-	-	-	-	-	-	-	-	15579	-	-	-
-	-	-	400.1	-	-	-	-	4043.5	-	-	-
-	-	-	-	-	-	-	1068	-	-	-	-
-	-	-	-	-	8562	-	-	-	-	-	-
12617	-	-	-	-	-	-	-	-	-	7602	-
-	-	2773	-	-	-	-	-	-	-	2155	-
-	-	-	-	-	-	-	-	-	-	-	-
8449.2	-	4680	-	-	-	-	-	-	11255	-	-
-	-	-	-	-	1126	-	-	-	-	-	-
-	-	-	2564	-	-	-	964.2	-	-	1726	-
-	-	-	1128	-	-	910.7	-	-	1283.8	-	-
-	-	-	1700	-	-	-	-	-	-	-	-
-	-	-	-	-	-	-	-	-	-	1742	-
-	-	-	-	-	-	-	4711	-	-	-	-
-	38198	-	-	-	-	-	-	-	-	-	-
44679	-	-	-	-	-	-	-	-	-	-	-
-	-	-	-	8449	-	--	-	-	12431	-	-
-	-	-	-	-	-	-	-	-	-	-	5818
-	-	-	-	-	-	-	-	-	-	-	8056
6897.6	-	-	-	-	-	4186	-	-	-	-	1258
-	-	-	-	-	640.6	-	-	-	-	-	-
-	-	-	-	-	-	-	-	2385	-	-	-
-	-	-	-	-	-	6924	-	-	-	1908	-

M46	M45	M44	M43	M42	M41	M40	M39	M38	M37	M36	M35
0.69	0.68	0.67	0.66	0.65	0.64	0.63	0.62	0.61	0.59	0.58	0.57
-	-	-	-	-	3263.9	-	-	-	3320.3	-	-
-	-	-	-	-	-	1213.3	-	-	3572.9	-	-
-	-	2727.6	-	-	-	-	1464.2	-	-	-	934.1
-	12084	-	-	-	-	-	3453.5	-	2660.9	-	-
-	-	-	-	-	18552	-	-	-	-	11841	-
-	-	13308	-	-	-	-	33593	-	-	-	-
-	-	-	-	-	5021.4	-	-	-	-	-	1502
9980	-	-	-	-	5823.7	-	-	-	-	8350.1	-
-	-	5862.2	-	-	-	-	3735	-	-	-	-
-	-	-	-	-	2262.6	-	-	-	-	1438	-
-	-	-	3967	-	-	-	-	-	-	-	2320
-	-	-	-	-	-	-	1767.7	-	-	3031.3	-
-	-	-	-	-	6468.4	-	-	-	-	9610.7	-
-	3524	-	-	-	4604.5	-	-	-	-	6510.1	-
4047	-	-	-	-	2615.8	-	-	-	-	-	5942
-	-	48702	-	-	-	28262	-	-	-	-	-
-	-	48310	-	-	-	-	29314	-	-	-	-
-	-	6667.9	-	-	-	-	-	13663	-	-	4333
-	-	-	-	-	14665	-	-	-	12288	-	-
-	-	-	-	-	17199	-	-	-	16124	-	-
-	-	-	-	3589	-	-	-	-	-	-	-
-	-	-	-	-	-	-	-	-	-	-	-
-	-	9864.1	-	-	-	-	15841	-	-	-	-
-	-	3039.4	-	-	-	-	-	-	-	7637.6	-

M58	M57	M56	M55	M54	M53	M52	M51	M50	M49	M48	M47
0.84	0.83	0.81	0.79	0.77	0.76	0.75	0.74	0.73	0.72	0.71	0.7
-	-	-	-	-	-	-	7161	-	-	-	-
-	-	-	-	-	-	-	-	3092	-	-	3923.8
-	-	-	-	-	-	-	-	-	-	4284	-
-	-	-	-	-	7423.3	-	-	-	-	-	-
-	-	-	-	29910	-	-	-	-	-	-	49632
-	7990.3	-	-	-	-	-	-	-	7274	-	-
-	-	-	-	-	-	-	-	2024	-	-	-
9910.7	-	-	-	-	-	-	-	-	-	-	-
-	6312	-	-	7144	-	-	-	-	-	-	-
6163.6	-	-	-	-	-	-	-	2572	-	-	-
9455	-	-	-	-	-	-	2899	-	-	-	3561.1
-	7949.6	-	-	-	-	2047	-	-	-	-	4798.1
-	-	-	-	-	-	-	-	2261	-	-	-
10810	-	-	-	-	-	-	-	-	-	-	-
-	8334.9	-	-	-	-	2410	-	-	-	-	-
-	34312	-	7622	-	-	10119	-	-	-	-	-
35377	-	-	8840	-	11314	-	-	-	-	-	-
25719	-	-	-	6629	-	-	-	-	5835	-	-
-	-	8048	-	-	-	-	7502	-	-	-	7028.9
-	-	9462	5945	-	-	-	7754	-	-	-	9090.5
-	8489.1	-	-	-	-	3636	-	-	-	4314	-
-	-	-	-	-	19105	-	-	-	-	-	-
-	-	-	-	-	-	-	-	-	-	6200	-
-	-	-	-	4786	-	-	-	-	-	5489	-

M59	0.85	-	-	-	6273	7852	-	-	-	-	-	-	-	-	-	-	-	-	-	-	-	-	-	-	-
M60	0.86	-	-	-	-	-	-	-	-	-	-	4771	8717	-	4243	8884	-	-	-	-	-	-	-	-	-
M61	0.87	16259	19664	21336	5176.7	6547.1	-	9136.8	4819.6	-	3205.9	-	-	9198.3	-	-	-	-	-	13405	15044	-	-	22347	-
M62	0.88	-	-	-	-	-	9589.5	-	-	9601.5	-	-	-	-	-	-	-	-	-	-	-	10495	24722	-	26524
M63	0.96	-	-	240	802	-	-	-	-	-	-	-	-	-	-	-	-	-	-	-	-	-	-	-	-
**Total Number of Metabolites**		**9**	**13**	**12**	**12**	**10**	**11**	**11**	**11**	**14**	**7**	**11**	**11**	**11**	**9**	**11**	**8**	**6**	**8**	**11**	**12**	**13**	**3**	**8**	**8**

Table S1 B: TLC profile of all three extract of different plant materials scanned at 366 nm.

Plant		*P. emblica*			*P. nigrum*			*T. cordifolia*			*W. somnifera*			*A. indica*			*C. longa*			*O. sanctum*			*A. millefolium*		
Extract		WE	EE	HEE	WE	EE	HEE	WE	EE	HEE	WE	EE	HEE	WE	EE	HEE	WE	EE	HEE	WE	EE	HEE	WE	EE	HEE
Metabolites	R_f																								
C1	0.02	-	397.7	301.4	-	822.8	-	-	-	1376	-	-	-	-	-	-	-	-	-	-	2452	-	-	600.7	-
C2	0.04	-	-	-	-	-	-	116.1	296.6	-	-	-	-	-	-	471.1	142.9	-	-	2437	-	1766	-	-	327.3
C3	0.05	437.8	1204	-	-	-	-	-	-	-	-	-	-	-	-	-	-	-	-	-	-	-	-	-	-
C4	0.08	318	967	-	-	-	-	179	596	-	-	-	-	-	-	-	-	-	-	-	-	-	-	-	-
C5	0.1	-	136.2	-	209.3	-	-	-	-	-	-	-	-	-	-	366.6	-	-	-	4420	4913	-	-	282.8	-
C6	0.11	-	-	-	-	-	-	-	-	-	-	-	-	-	-	-	-	-	-	-	-	565	-	-	-
C7	0.19	-	1789	-	-	-	-	-	-	-	-	-	-	-	-	-	-	-	-	433.8	538.7	-	-	-	-
C8	0.22	-	-	20593	-	-	-	-	-	-	-	-	-	-	-	-	527.4	-	-	-	-	-	-	-	-
C9	0.23	-	1424	-	-	-	-	-	-	-	-	-	-	-	-	-	-	-	-	-	-	-	-	-	-

C22	C21	C20	C19	C18	C17	C16	C15	C14	C13	C12	C11	C10
0.48	0.47	0.46	0.43	0.42	0.41	0.39	0.37	0.36	0.34	0.29	0.27	0.24
6378	-	-	-	-	-	11094	-	-	-	-	500	-
6773	-	-	-	-	-	17491	-	-	-	-	-	-
770.3	-	-	-	1024	-	-	-	3448	-	-	-	-
-	-	-	-	-	-	-	-	-	-	-	-	-
-	-	1391	-	2182	-	-	-	-	-	-	-	-
-	-	2665	-	-	-	-	-	-	-	-	-	-
-	-	-	-	-	-	-	-	-	-	-	-	-
-	-	-	-	-	-	-	-	-	-	-	-	-
-	-	2876	-	-	947	-	2051	-	-	-	-	805
-	-	-	-	-	-	-	-	-	-	-	-	-
-	-	-	-	-	-	-	-	-	-	-	-	-
-	-	-	-	-	-	-	-	-	-	-	-	-
859.6	-	-	-	-	-	-	-	-	-	-	-	-
-	-	-	-	-	-	-	-	-	-	-	-	-
-	-	667.1	-	-	-	-	-	-	-	-	-	-
-	-	-	-	-	-	-	-	-	-	-	-	-
-	-	-	-	-	-	-	-	-	-	-	-	-
-	17034	-	-	-	-	-	-	-	-	-	-	-
-	-	-	6774	-	-	-	-	-	-	-	-	-
-	-	-	7963	-	-	-	815.7	-	-	-	-	-
-	-	-	496.6	-	-	-	-	-	-	-	3556	-
-	-	-	-	-	-	-	-	-	-	-	-	-
2210	-	-	-	-	-	650.6	-	-	-	-	-	-
-	-	-	-	-	-	-	-	-	501	511	-	-

C34	C33	C32	C31	C30	C29	C28	C27	C26	C25	C24	C23
0.61	0.6	0.59	0.58	0.56	0.55	0.54	0.53	0.52	0.51	0.5	0.49
-	-	4362	-	-	-	-	-	-	-	-	-
-	-	2022.3	-	-	-	-	-	-	-	-	-
-	-	-	-	-	-	-	-	-	-	-	-
-	-	985.7	-	-	-	-	-	-	-	-	-
-	-	-	5903	-	-	-	-	-	2766	-	-
-	-	-	-	17335	-	-	-	-	-	1229	-
-	-	-	-	-	462	-	-	-	-	-	-
-	-	-	2300	-	-	-	-	-	-	-	-
-	-	-	-	-	-	5460	-	-	-	-	2647
-	-	-	629.4	-	-	-	-	-	-	-	-
-	1594	-	-	-	-	-	-	-	-	-	-
-	-	-	452.3	-	-	-	-	-	-	371.3	-
-	-	-	2796	-	-	-	-	-	-	-	-
-	-	-	2214	-	-	-	-	-	-	-	-
-	-	-	-	1079.8	-	-	673	-	-	-	1008
90822	-	-	-	-	-	-	-	-	-	-	-
-	-	-	-	-	-	-	-	-	-	-	-
24783	-	-	-	-	-	-	-	12570	-	-	-
-	-	12348	-	-	-	-	-	-	-	-	-
-	-	14800	-	-	-	-	-	-	-	-	-
1062.6	-	-	-	4843.6	-	-	-	-	-	3782	-
-	-	-	-	-	-	-	-	-	-	-	-
-	-	-	-	-	-	-	-	-	-	-	-
-	-	-	4055	-	-	-	-	-	9963	-	-

C45	C44	C43	C42	C41	C40	C39	C38	C37	C36	C35
0.75	0.74	0.73	0.71	0.7	0.69	0.68	0.67	0.66	0.64	0.62
-	-	-	-	2518.3	-	-	-	-	1706.7	-
-	-	3129	-	1902	-	-	-	-	-	-
-	-	-	1798	-	-	-	900	-	-	982.3
-	-	-	-	-	-	15228	-	-	-	2511.2
-	-	-	-	47531	-	-	-	-	18828	-
-	-	-	-	-	-	-	11323	-	-	34922
-	-	-	-	2002.9	-	-	-	-	-	-
-	-	-	-	-	8567	-	-	-	1934.3	-
-	-	769.8	-	-	1166	-	-	-	-	2432.6
-	-	-	-	2546.3	-	-	-	-	-	-
-	1983	-	-	967	-	-	-	1233	-	-
-	-	-	-	1556.5	-	-	-	965.5	-	861.1
-	-	-	-	2047.2	-	-	-	-	1974.9	-
1446.1	-	-	2198	-	-	-	-	-	1323.9	-
-	-	-	-	-	1483	-	-	-	1300	-
-	-	-	-	-	-	-	66994	-	-	-
12712	-	-	-	-	-	53910	-	-	-	96243
-	-	-	-	1182.6	-	-	-	-	-	-
-	-	-	6747	-	-	-	5965.8	-	12531	-
-	5172	-	-	-	-	-	-	-	23174	-
-	-	-	-	1587.3	-	-	1474.7	-	-	-
-	-	-	-	-	-	-	-	-	-	-
-	-	-	5936	-	-	-	-	6526	-	14997
-	-	-	2657	-	-	-	2769.5	-	-	-

C46	0.76	867	-	-	-	-	-	-	-	-	-	-	-	-	-	-	-	-	-	-	-	-	10281	-	-
C47	0.77	-	-	-	-	-	-	-	-	-	-	-	-	-	-	-	-	-	-	-	-	-	-	3295	-
C48	0.79	0	-	-	-	-	-	-	-	1454	-	-	-	-	-	-	-	-	-	-	-	-	-	-	5588
C49	0.81	0	-	-	-	2764.5	-	-	-	-	-	-	-	-	-	-	-	-	-	10389	10880	-	-	-	-
C50	0.83	-	-	-	-	-	-	-	-	-	-	-	-	-	-	-	2664	-	-	-	-	-	-	-	-
C51	0.84	-	-	-	-	-	-	-	-	-	1899	-	-	1963	-	-	-	-	-	-	-	-	-	-	-
C52	0.85	-	-	-	2464.3	-	-	1852.3	-	-	-	-	-	-	-	-	-	3066.5	-	10194	-	-	-	-	-
C53	0.86	-	-	-	-	-	2840	-	3411.7	3048.9	-	2989.3	2478.5	-	2434.8	2774.5	-	-	3049	-	10595	-	7263.6	-	-
C54	0.87	4081	4551	4644	-	3747	-	-	-	-	-	-	-	-	-	-	-	-	-	-	-	-	-	8526	-
C55	0.88	-	-	-	-	-	-	-	-	-	-	-	-	-	-	-	-	-	-	-	-	3559	-	-	-
C56	0.96	-	-	-	437	-	-	-	-	-	-	-	-	-	-	-	-	-	-	-	-	-	-	-	-
**Total Number of Metabolites**		**10**	**12**	**9**	**6**	**9**	**6**	**5**	**6**	**12**	**3**	**5**	**6**	**5**	**5**	**9**	**5**	**4**	**5**	**10**	**10**	**10**	**2**	**9**	**8**

Table S2: Correlation matrix (Pearson n) of variables.

**Variables**	**(*P. emblica*-WE)**	**(*P. emblica*-EE)**	**(*P. emblica*-HEE)**	**(*P. nigrum*-WE)**	**(*P. nigrum*-EE)**	**(*P. nigrum*-HEE)**	**(*T. cordifolia*-WE)**	**(*T. cordifolia*-EE)**	**(*T. cordifolia*-HEE)**	**(*W. somnifera*-WE)**	**(*W. somnifera*-EE)**	**(*W. somnifera*-HEE)**	**(*A. indica*-WE)**	**(*A. indica*-EE)**	**(*A. indica*-HEE)**	**(*C. longa*-WE)**	**(*C. longa*-EE)**	**(*C. longa*-HEE)**	**(*O. sanctum*-WE)**	**(*O. sanctum*-EE)**	**(*O. sanctum*-HEE)**	**(*A. millefolium*-WE)**	**(*A. millefolium*-EE)**	**(*A. millefolium*-HEE)**
***P. emblica*-WE**	**1**	0.843	0.256	0.061	0.059	-0.08	0.354	0.081	-0.07	0.18	-0.03	-0.07	0.304	-0.04	-0.06	-0.07	-0.07	-0.08	0.284	0.263	-0.09	-0.04	0.36	-0.07
***P. emblica*-EE**		**1**	0.238	0.059	0.071	-0.08	0.394	0.052	-0.11	0.147	-0.05	-0.04	0.258	-0.08	-0.08	-0.06	-0.07	-0.08	0.166	0.152	-0.1	-0.05	0.362	-0.07
***P. emblica*-HEE**			**1**	0.054	-0.02	0.009	0.33	0.052	-0.07	0.126	-0.04	-0.06	0.232	-0.06	-0.07	-0.02	-0.01	-0.06	0.102	0.076	-0.05	-0.04	0.346	-0.03
***P. nigrum*-WE**				**1**	-0.02	0.089	0.147	-0.02	-0.06	0.025	-0.07	-0.03	0.073	0.06	-0.06	-0.06	0.354	-0.08	0.079	0.043	-0.09	0.145	0.196	-0.06
***P. nigrum*-EE**					**1**	-0.07	0.107	0.115	0.027	0.26	0.139	0.256	0.303	0.103	-0.02	-0.06	-0.06	-0	0.249	0.286	-0.04	-0.04	-0.01	0.052
***P. nigrum*-HEE**						**1**	-0.04	-0.07	0.343	-0.03	-0.05	0.135	-0.05	-0.05	0.03	0.162	0.67	-0.02	-0.07	-0.09	0.261	0.091	0.534	0.126
***T. cordifolia*-WE**							**1**	0.131	-0.09	0.3	-0.01	-0.04	0.438	-0.04	-0.02	0.107	-0.06	-0.05	0.257	0.183	-0.07	-0.04	0.45	-0.05
***T. cordifolia*-EE**								**1**	-0.06	0.555	0.32	0.052	0.5	0.622	0.184	-0.06	0.066	0.211	0.144	0.187	-0.1	-0.01	0.081	0.05
***T. cordifolia*-HEE**									**1**	-0.09	-0.08	0.171	-0.09	-0.07	0.089	0.085	0.251	0.125	-0.14	-0.1	0.396	0.291	0.1	0.365

***W. somnifera*-WE**										**1**	0.489	0.009	0.517	0.647	-0.03	-0.06	0.138	0.39	0.167	0.148	-0.06	-0.04	0.182	-0.01
***W. somnifera*-EE**											**1**	0.327	-0.05	0.613	0.262	-0.07	0.122	0.357	-0.01	0.061	-0.1	0	-0.06	-0.06
***W. somnifera*-HEE**												**1**	0.099	0.249	0.676	0.101	0.01	-0.04	-0.03	0.018	0.238	-0.01	0.026	0.007
***A. indica*-WE**													**1**	0.381	0.007	-0.06	-0.06	-0.05	0.349	0.339	-0.08	-0.05	0.319	0.113
***A. indica*-EE**														**1**	0.181	-0.06	0.14	0.344	0.091	0.112	-0.09	-0.01	-0.05	0.082
***A. indica*-HEE**															**1**	0.086	-0.07	-0.04	-0	0.052	0.204	-0.01	-0.08	-0.07
***C. longa*-WE**																**1**	0.082	0.355	0	-0.07	0.135	-0.04	0.043	0.025
***C. longa*-EE**																	**1**	0.133	-0.08	-0.08	0.043	0.008	0.45	-0.02
***C. longa*-HEE**																		**1**	-0.11	-0.09	-0.07	-0.03	-0.04	-0.03
***O. sanctum*-WE**																			**1**	0.86	-0.09	-0.06	0.178	-0.06
***O. sanctum*-EE**																				**1**	-0.11	-0.01	0.124	-0.09
***O. sanctum*-HEE**																					**1**	0.367	-0.05	0.501

***A. millefolium*-WE**																						**1**	-0.05	0.589
***A. millefolium*-EE**																							**1**	0.01
***A. millefolium*-HEE**																								**1**

Table S3: Eigenvalues of variables from principle component analysis (PCA).

	F1	F2	F3	F4	F5	F6	F7	F8	F9	F10	F11	F12
**Eigenvalue**	3.9	2.96	2.63	2.04	1.71	1.52	1.3	1.08	1.01	0.92	0.83	0.66
**Variability (%)**	16.24	12.32	10.98	8.491	7.114	6.342	5.431	4.502	4.222	3.848	3.457	2.734
**Cumulative %**	16.24	28.57	39.54	48.03	55.15	61.49	66.92	71.42	75.64	79.49	82.95	85.68

	F13	F14	F15	F16	F17	F18	F19	F20	F21	F22	F23	F24
**Eigenvalue**	0.59	0.55	0.43	0.37	0.32	0.24	0.21	0.19	0.16	0.14	0.13	0.1
**Variability (%)**	2.477	2.287	1.777	1.554	1.35	0.989	0.886	0.795	0.685	0.563	0.527	0.429
**Cumulative %**	88.16	90.45	92.22	93.78	95.13	96.12	97	97.8	98.48	99.05	99.57	100

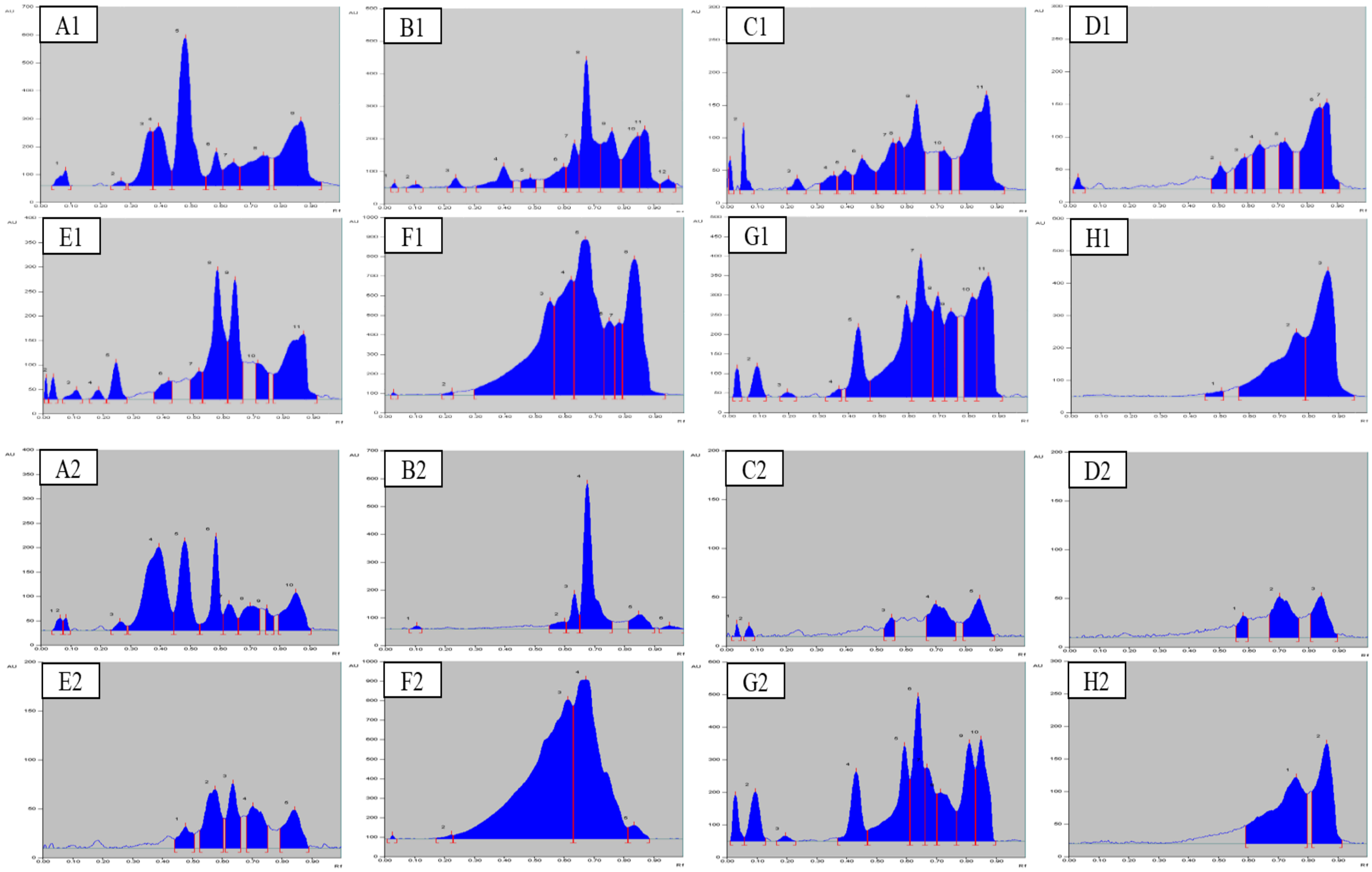

Figure S1: Developed thin layer chromatogram of Water Extracts (WE) of *P. emblica* (A1), *P. nigrum* (B1), *T. cordifolia* (C1), *W. somnifera* (D1), *A. indica* (E1), *C. longa* (F1), *O. sanctum* (G1), *A. millefolium* (H1) at 254 nm and *P. emblica* (A2), *P. nigrum* (B2), *T. cordifolia* (C2), *W. somnifera* (D2), *A. indica* (E2), *C. longa* (F2), *O. sanctum* (G2), *A. millefolium* (H2) at 366 nm.

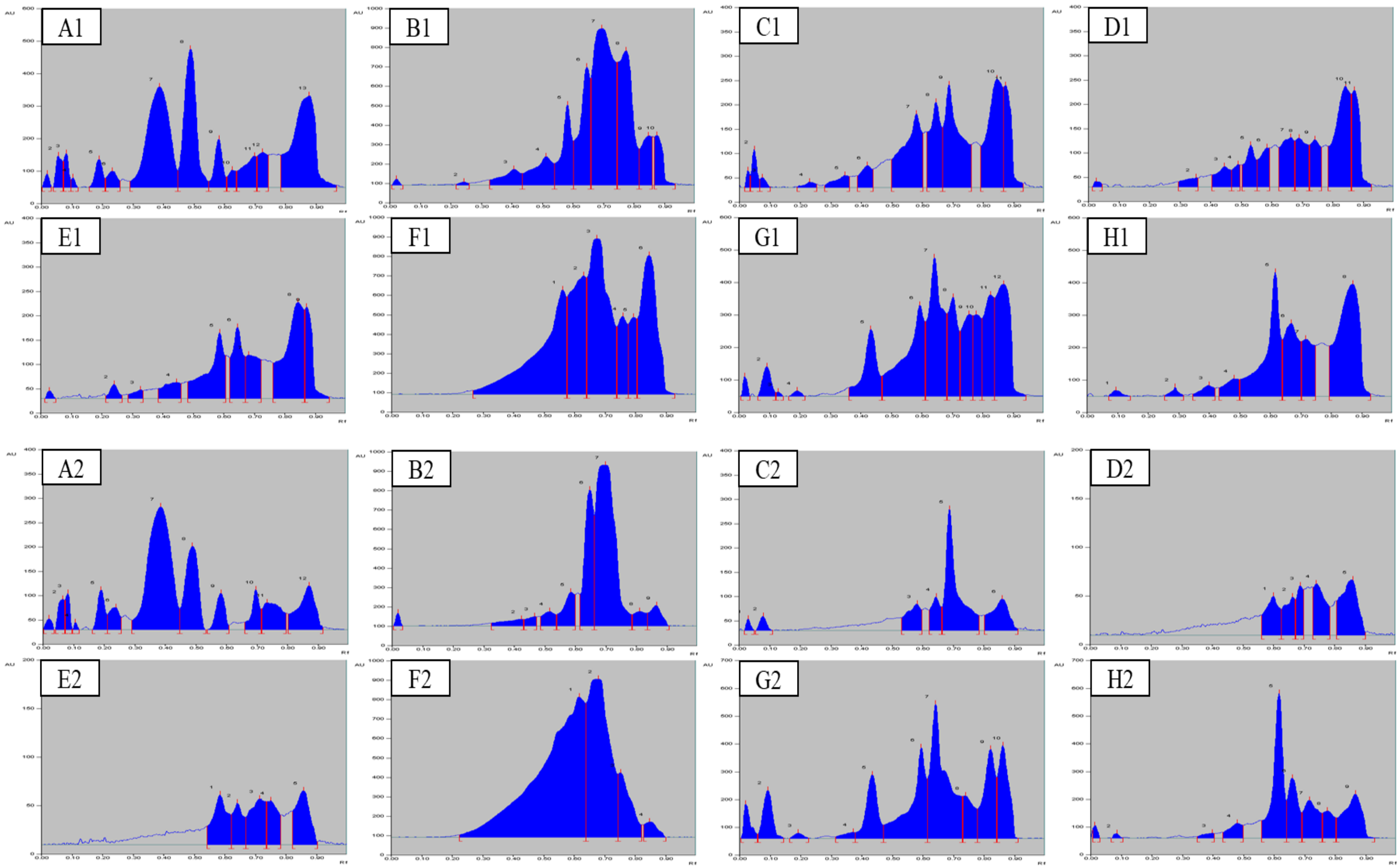

Figure S2: Developed thin layer chromatogram of Ethanolic Extracts (EE) of *P. emblica* (A1), *P. nigrum* (B1), *T. cordifolia* (C1), *W. somnifera* (D1), *A. indica* (E1), *C. longa* (F1), *O. sanctum* (G1), *A. millefolium* (H1) at 254 nm and *P. emblica* (A2), *P. nigrum* (B2), *T. cordifolia* (C2), *W. somnifera* (D2), *A. indica* (E2), *C. longa* (F2), *O. sanctum* (G2), *A. millefolium* (H2) at 366 nm.

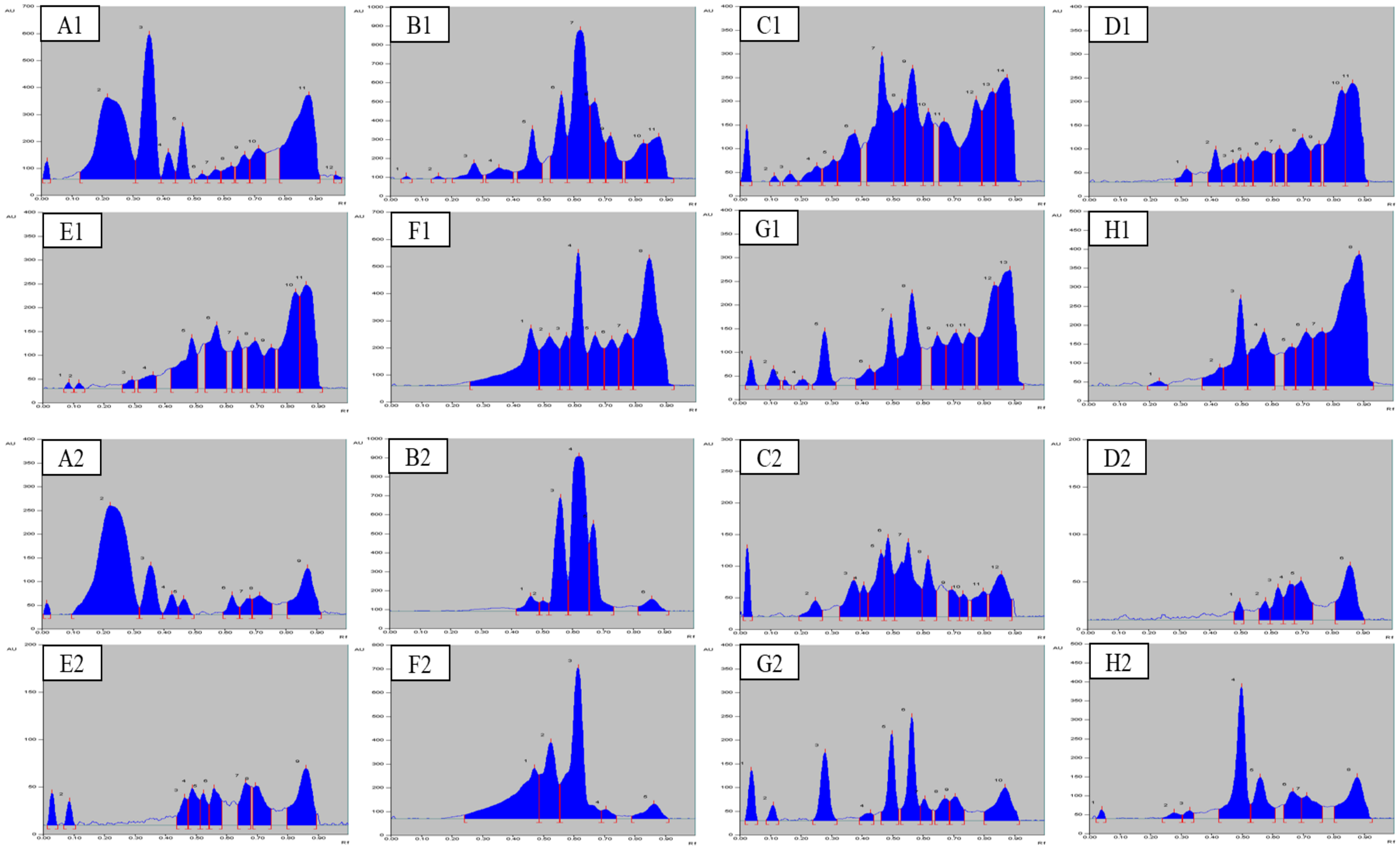

Figure S3: Developed thin layer chromatogram of Hydroethanolic Extracts (HEE) of *P. emblica* (A1), *P. nigrum* (B1), *T. cordifolia* (C1), *W. somnifera* (D1), *A. indica* (E1), *C. longa* (F1), *O. sanctum* (G1), *A. millefolium* (H1) at 254 nm and *P. emblica* (A2), *P. nigrum* (B2), *T. cordifolia* (C2), *W. somnifera* (D2), *A. indica* (E2), *C. longa* (F2), *O. sanctum* (G2), *A. millefolium* (H2) at 366 nm.
