# Supplementary material for: TLC-Based Metabolite Profiling and Bioactivity-Based Scientific Validation for Use of Water Extracts in AYUSH Formulations
Source: Evid Based Complement Alternat Med. 2021 Dec 31;2021:2847440. doi: 10.1155/2021/2847440 (PMC8741349; doi:10.1155/2021/2847440)
Supplement: Supplementary Materials — Supplementary Table S1 A: TLC profile of all three extracts of different plant materials scanned at 254 nm. Supplementary Table S1 B: TLC profile of all three extracts of different plant materials scanned at 366 nm. Supplementary Table S2: correlation matrix (Pearson n) of variables. Supplementary Table S3: eigenvalues of variables from principal component analysis (PCA). Figure S1: developed thin-layer chromatogram of water extract (WE) of P. emblica (A1), P. nigrum (B1), T. cordifolia (C1), W. somnifera (D1), A. indica (E1), C. longa (F1), O. sanctum (G1), and A. millefolium (H1) at 254 nm and P. emblica (A2), P. nigrum (B2), T. cordifolia (C2), W. somnifera (D2), A. indica (E2), C. longa (F2), O. sanctum (G2), and A. millefolium (H2) at 366 nm. Figure S2: developed thin-layer chromatogram of ethanolic extract (EE) of P. emblica (A1), P. nigrum (B1), T. cordifolia (C1), W. somnifera (D1), A. indica (E1), C. longa (F1), O. sanctum (G1), and A. millefolium (H1) at 254 nm and P. emblica (A2), P. nigrum (B2), T. cordifolia (C2), W. somnifera (D2), A. indica (E2), C. longa (F2), O. sanctum (G2), and A. millefolium (H2) at 366 nm. Figure S3: developed thin-layer chromatogram of hydroethanolic extract (HEE) of P. emblica (A1), P. nigrum (B1), T. cordifolia (C1), W. somnifera (D1), A. indica (E1), C. longa (F1), O. sanctum (G1), and A. millefolium (H1) at 254 nm and P. emblica (A2), P. nigrum (B2), T. cordifolia (C2), W. somnifera (D2), A. indica (E2), C. longa (F2), O. sanctum (G2), and A. millefolium (H2) at 366 nm. [file 2847440.f1.zip › 2847440.f1/Table S1 A (1).pdf]

Supplementary Table S1 A: TLC profile of all three extract of different plant materials scanned at 254 nm.

Plant		*P. emblica*			*P. nigrum*			*T. cordifolia*			*W. somnifera*			*A. indica*			*C. longa*			*O. sanctum*			*A. millefolium*		
Extract		WE	EE	HEE	WE	EE	HEE	WE	EE	HEE	WE	EE	HEE	WE	EE	HEE	WE	EE	HEE	WE	EE	HEE	WE	EE	HEE
Metabolites	Rf																								
M1	0.02	-	567.1	813.1	-	459.5	-	394.2	-	1555	-	-	-	239.5	-	-	-	-	-	-	785.1	-	-	-	-
M2	0.04	-	-	-	159	-	-	-	348	-	286	225	-	539	249	-	110	-	-	965	-	831	-	-	-
M3	0.05	-	1862	-	-	-	203.8	1085	1152	-	-	-	-	-	-	-	-	-	-	-	-	-	-	-	-
M4	0.08	1277	1491	-	-	-	-	-	362	-	-	-	-	-	-	-	-	-	-	-	-	-	-	-	-
M5	0.1	-	336	-	276.6	-	-	-	-	-	-	-	-	-	-	160.6	-	-	-	1943	2307	-	-	533.3	-
M6	0.11	-	-	-	-	-	-	-	-	183	-	-	-	481	-	178	-	-	-	-	-	735	-	-	-
M7	0.13	-	-	-	-	-	-	-	-	-	-	-	-	-	-	-	-	-	-	-	177	-	-	-	-
M8	0.16	-	-	-	-	-	264	-	-	376	-	-	-	-	-	-	-	-	-	-	-	157	-	-	-
M9	0.19	-	1935	-	-	-	-	-	-	-	-	-	-	402.8	-	-	-	-	-	322.7	392.3	-	-	-	-

M22	M21	M20	M19	M18	M17	M16	M15	M14	M13	M12	M11	M10
0.42	0.4	0.39	0.37	0.36	0.35	0.34	0.32	0.29	0.27	0.24	0.23	0.22
-	-	8004.5	5879	-	-	-	-	-	417.4	-	-	-
-	-	20919	-	-	-	-	-	-	-	-	1500	-
2304	-	-	-	-	19508	-	-	-	-	-	-	26603
-	2602	-	-	-	-	-	-	-	-	679.7	-	-
4604	-	-	-	-	-	-	-	-	-	416.5	-	-
-	-	-	-	-	3116.2	-	-	-	2668	-	-	-
-	1019	-	-	791	-	-	-	-	-	-	452	-
1526	-	-	-	-	-	1094	-	-	-	-	354.1	-
-	-	-	4767	-	-	-	1494	-	-	1185	-	-
-	-	-	-	-	-	-	-	-	-	-	-	-
-	-	-	-	-	818.4	-	-	-	-	-	-	-
-	1544	-	-	-	-	-	800	-	-	-	-	-
1439	-	-	-	-	-	-	-	-	-	1706	-	-
-	-	-	-	-	-	-	546	-	-	719.5	-	-
-	-	-	-	1212	-	-	-	490	-	-	-	-
-	-	-	-	-	-	-	-	-	-	-	-	340
-	-	-	-	-	-	-	-	-	-	-	-	-
-	-	-	-	-	-	-	-	-	-	-	-	-
-	-	-	496.8	-	-	-	-	-	-	-	-	-
-	-	-	-	-	-	-	-	-	-	-	-	-
-	-	-	-	-	-	-	-	-	2785	-	-	310.5
-	-	-	-	-	-	-	-	-	-	-	-	-
-	-	1335.1	-	-	-	-	-	626	-	-	-	-
-	-	-	-	-	-	-	-	-	-	-	376.3	-

M34	M33	M32	M31	M30	M29	M28	M27	M26	M25	M24	M23
0.56	0.55	0.54	0.53	0.52	0.51	0.5	0.49	0.48	0.47	0.44	0.43
-	-	-	-	-	-	-	-	20908	-	-	-
-	-	-	-	-	-	-	-	15579	-	-	-
-	-	-	400.1	-	-	-	-	4043.5	-	-	-
-	-	-	-	-	-	-	1068	-	-	-	-
-	-	-	-	-	8562	-	-	-	-	-	-
12617	-	-	-	-	-	-	-	-	-	7602	-
-	-	2773	-	-	-	-	-	-	-	2155	-
-	-	-	-	-	-	-	-	-	-	-	-
8449.2	-	4680	-	-	-	-	-	-	11255	-	-
-	-	-	-	-	1126	-	-	-	-	-	-
-	-	-	2564	-	-	-	964.2	-	-	1726	-
-	-	-	1128	-	-	910.7	-	-	1283.8	-	-
-	-	-	1700	-	-	-	-	-	-	-	-
-	-	-	-	-	-	-	-	-	-	1742	-
-	-	-	-	-	-	-	4711	-	-	-	-
-	38198	-	-	-	-	-	-	-	-	-	-
44679	-	-	-	-	-	-	-	-	-	-	-
-	-	-	-	8449	-	--	-	-	12431	-	-
-	-	-	-	-	-	-	-	-	-	-	5818
-	-	-	-	-	-	-	-	-	-	-	8056
6897.6	-	-	-	-	-	4186	-	-	-	-	1258
-	-	-	-	-	640.6	-	-	-	-	-	-
-	-	-	-	-	-	-	-	2385	-	-	-
-	-	-	-	-	-	6924	-	-	-	1908	-

M46	M45	M44	M43	M42	M41	M40	M39	M38	M37	M36	M35
0.69	0.68	0.67	0.66	0.65	0.64	0.63	0.62	0.61	0.59	0.58	0.57
-	-	-	-	-	3263.9	-	-	-	3320.3	-	-
-	-	-	-	-	-	1213.3	-	-	3572.9	-	-
-	-	2727.6	-	-	-	-	1464.2	-	-	-	934.1
-	12084	-	-	-	-	-	3453.5	-	2660.9	-	-
-	-	-	-	-	18552	-	-	-	-	11841	-
-	-	13308	-	-	-	-	33593	-	-	-	-
-	-	-	-	-	5021.4	-	-	-	-	-	1502
9980	-	-	-	-	5823.7	-	-	-	-	8350.1	-
-	-	5862.2	-	-	-	-	3735	-	-	-	-
-	-	-	-	-	2262.6	-	-	-	-	1438	-
-	-	-	3967	-	-	-	-	-	-	-	2320
-	-	-	-	-	-	-	1767.7	-	-	3031.3	-
-	-	-	-	-	6468.4	-	-	-	-	9610.7	-
-	3524	-	-	-	4604.5	-	-	-	-	6510.1	-
4047	-	-	-	-	2615.8	-	-	-	-	-	5942
-	-	48702	-	-	-	28262	-	-	-	-	-
-	-	48310	-	-	-	-	29314	-	-	-	-
-	-	6667.9	-	-	-	-	-	13663	-	-	4333
-	-	-	-	-	14665	-	-	-	12288	-	-
-	-	-	-	-	17199	-	-	-	16124	-	-
-	-	-	-	3589	-	-	-	-	-	-	-
-	-	-	-	-	-	-	-	-	-	-	-
-	-	9864.1	-	-	-	-	15841	-	-	-	-
-	-	3039.4	-	-	-	-	-	-	-	7637.6	-

M58	M57	M56	M55	M54	M53	M52	M51	M50	M49	M48	M47
0.84	0.83	0.81	0.79	0.77	0.76	0.75	0.74	0.73	0.72	0.71	0.7
-	-	-	-	-	-	-	7161	-	-	-	-
-	-	-	-	-	-	-	-	3092	-	-	3923.8
-	-	-	-	-	-	-	-	-	-	4284	-
-	-	-	-	-	7423.3	-	-	-	-	-	-
-	-	-	-	29910	-	-	-	-	-	-	49632
-	7990.3	-	-	-	-	-	-	-	7274	-	-
-	-	-	-	-	-	-	-	2024	-	-	-
9910.7	-	-	-	-	-	-	-	-	-	-	-
-	6312	-	-	7144	-	-	-	-	-	-	-
6163.6	-	-	-	-	-	-	-	2572	-	-	-
9455	-	-	-	-	-	-	2899	-	-	-	3561.1
-	7949.6	-	-	-	-	2047	-	-	-	-	4798.1
-	-	-	-	-	-	-	-	2261	-	-	-
10810	-	-	-	-	-	-	-	-	-	-	-
-	8334.9	-	-	-	-	2410	-	-	-	-	-
-	34312	-	7622	-	-	10119	-	-	-	-	-
35377	-	-	8840	-	11314	-	-	-	-	-	-
25719	-	-	-	6629	-	-	-	-	5835	-	-
-	-	8048	-	-	-	-	7502	-	-	-	7028.9
-	-	9462	5945	-	-	-	7754	-	-	-	9090.5
-	8489.1	-	-	-	-	3636	-	-	-	4314	-
-	-	-	-	-	19105	-	-	-	-	-	-
-	-	-	-	-	-	-	-	-	-	6200	-
-	-	-	-	4786	-	-	-	-	-	5489	-

M59	0.85	-	-	-	6273	7852	-	-	-	-	-	-	-	-	-	-	-	-	-	-	-	-	-	-	-
M60	0.86	-	-	-	-	-	-	-	-	-	-	4771	8717	-	4243	8884	-	-	-	-	-	-	-	-	-
M61	0.87	16259	19664	21336	5176.7	6547.1	-	9136.8	4819.6	-	3205.9	-	-	9198.3	-	-	-	-	-	13405	15044	-	-	22347	-
M62	0.88	-	-	-	-	-	9589.5	-	-	9601.5	-	-	-	-	-	-	-	-	-	-	-	10495	24722	-	26524
M63	0.96	-	-	240	802	-	-	-	-	-	-	-	-	-	-	-	-	-	-	-	-	-	-	-	-
**Total Number of Metabolites**		**9**	**13**	**12**	**12**	**10**	**11**	**11**	**11**	**14**	**7**	**11**	**11**	**11**	**9**	**11**	**8**	**6**	**8**	**11**	**12**	**13**	**3**	**8**	**8**
