# Supplementary material for: TLC-Based Metabolite Profiling and Bioactivity-Based Scientific Validation for Use of Water Extracts in AYUSH Formulations
Source: Evid Based Complement Alternat Med. 2021 Dec 31;2021:2847440. doi: 10.1155/2021/2847440 (PMC8741349; doi:10.1155/2021/2847440)
Supplement: Supplementary Materials — Supplementary Table S1 A: TLC profile of all three extracts of different plant materials scanned at 254 nm. Supplementary Table S1 B: TLC profile of all three extracts of different plant materials scanned at 366 nm. Supplementary Table S2: correlation matrix (Pearson n) of variables. Supplementary Table S3: eigenvalues of variables from principal component analysis (PCA). Figure S1: developed thin-layer chromatogram of water extract (WE) of P. emblica (A1), P. nigrum (B1), T. cordifolia (C1), W. somnifera (D1), A. indica (E1), C. longa (F1), O. sanctum (G1), and A. millefolium (H1) at 254 nm and P. emblica (A2), P. nigrum (B2), T. cordifolia (C2), W. somnifera (D2), A. indica (E2), C. longa (F2), O. sanctum (G2), and A. millefolium (H2) at 366 nm. Figure S2: developed thin-layer chromatogram of ethanolic extract (EE) of P. emblica (A1), P. nigrum (B1), T. cordifolia (C1), W. somnifera (D1), A. indica (E1), C. longa (F1), O. sanctum (G1), and A. millefolium (H1) at 254 nm and P. emblica (A2), P. nigrum (B2), T. cordifolia (C2), W. somnifera (D2), A. indica (E2), C. longa (F2), O. sanctum (G2), and A. millefolium (H2) at 366 nm. Figure S3: developed thin-layer chromatogram of hydroethanolic extract (HEE) of P. emblica (A1), P. nigrum (B1), T. cordifolia (C1), W. somnifera (D1), A. indica (E1), C. longa (F1), O. sanctum (G1), and A. millefolium (H1) at 254 nm and P. emblica (A2), P. nigrum (B2), T. cordifolia (C2), W. somnifera (D2), A. indica (E2), C. longa (F2), O. sanctum (G2), and A. millefolium (H2) at 366 nm. [file 2847440.f1.zip › 2847440.f1/Table S1 B (1).pdf]

Supplementary Table S1 B: TLC profile of all three extract of different plant materials scanned at 366 nm.

Plant		*P. emblica*			*P. nigrum*			*T. cordifolia*			*W. somnifera*			*A. indica*			*C. longa*			*O. sanctum*			*A. millefolium*		
Extract		WE	EE	HEE	WE	EE	HEE	WE	EE	HEE	WE	EE	HEE	WE	EE	HEE	WE	EE	HEE	WE	EE	HEE	WE	EE	HEE
Metabolites	R_f																								
C1	0.02	-	397.7	301.4	-	822.8	-	-	-	1376	-	-	-	-	-	-	-	-	-	-	2452	-	-	600.7	-
C2	0.04	-	-	-	-	-	-	116.1	296.6	-	-	-	-	-	-	471.1	142.9	-	-	2437	-	1766	-	-	327.3
C3	0.05	437.8	1204	-	-	-	-	-	-	-	-	-	-	-	-	-	-	-	-	-	-	-	-	-	-
C4	0.08	318	967	-	-	-	-	179	596	-	-	-	-	-	-	-	-	-	-	-	-	-	-	-	-
C5	0.1	-	136.2	-	209.3	-	-	-	-	-	-	-	-	-	-	366.6	-	-	-	4420	4913	-	-	282.8	-
C6	0.11	-	-	-	-	-	-	-	-	-	-	-	-	-	-	-	-	-	-	-	-	565	-	-	-
C7	0.19	-	1789	-	-	-	-	-	-	-	-	-	-	-	-	-	-	-	-	433.8	538.7	-	-	-	-
C8	0.22	-	-	20593	-	-	-	-	-	-	-	-	-	-	-	-	527.4	-	-	-	-	-	-	-	-
C9	0.23	-	1424	-	-	-	-	-	-	-	-	-	-	-	-	-	-	-	-	-	-	-	-	-	-

C22	C21	C20	C19	C18	C17	C16	C15	C14	C13	C12	C11	C10
0.48	0.47	0.46	0.43	0.42	0.41	0.39	0.37	0.36	0.34	0.29	0.27	0.24
6378	-	-	-	-	-	11094	-	-	-	-	500	-
6773	-	-	-	-	-	17491	-	-	-	-	-	-
770.3	-	-	-	1024	-	-	-	3448	-	-	-	-
-	-	-	-	-	-	-	-	-	-	-	-	-
-	-	1391	-	2182	-	-	-	-	-	-	-	-
-	-	2665	-	-	-	-	-	-	-	-	-	-
-	-	-	-	-	-	-	-	-	-	-	-	-
-	-	-	-	-	-	-	-	-	-	-	-	-
-	-	2876	-	-	947	-	2051	-	-	-	-	805
-	-	-	-	-	-	-	-	-	-	-	-	-
-	-	-	-	-	-	-	-	-	-	-	-	-
-	-	-	-	-	-	-	-	-	-	-	-	-
859.6	-	-	-	-	-	-	-	-	-	-	-	-
-	-	-	-	-	-	-	-	-	-	-	-	-
-	-	667.1	-	-	-	-	-	-	-	-	-	-
-	-	-	-	-	-	-	-	-	-	-	-	-
-	-	-	-	-	-	-	-	-	-	-	-	-
-	17034	-	-	-	-	-	-	-	-	-	-	-
-	-	-	6774	-	-	-	-	-	-	-	-	-
-	-	-	7963	-	-	-	815.7	-	-	-	-	-
-	-	-	496.6	-	-	-	-	-	-	-	3556	-
-	-	-	-	-	-	-	-	-	-	-	-	-
2210	-	-	-	-	-	650.6	-	-	-	-	-	-
-	-	-	-	-	-	-	-	-	501	511	-	-

C34	C33	C32	C31	C30	C29	C28	C27	C26	C25	C24	C23
0.61	0.6	0.59	0.58	0.56	0.55	0.54	0.53	0.52	0.51	0.5	0.49
-	-	4362	-	-	-	-	-	-	-	-	-
-	-	2022.3	-	-	-	-	-	-	-	-	-
-	-	-	-	-	-	-	-	-	-	-	-
-	-	985.7	-	-	-	-	-	-	-	-	-
-	-	-	5903	-	-	-	-	-	2766	-	-
-	-	-	-	17335	-	-	-	-	-	1229	-
-	-	-	-	-	462	-	-	-	-	-	-
-	-	-	2300	-	-	-	-	-	-	-	-
-	-	-	-	-	-	5460	-	-	-	-	2647
-	-	-	629.4	-	-	-	-	-	-	-	-
-	1594	-	-	-	-	-	-	-	-	-	-
-	-	-	452.3	-	-	-	-	-	-	371.3	-
-	-	-	2796	-	-	-	-	-	-	-	-
-	-	-	2214	-	-	-	-	-	-	-	-
-	-	-	-	1079.8	-	-	673	-	-	-	1008
90822	-	-	-	-	-	-	-	-	-	-	-
-	-	-	-	-	-	-	-	-	-	-	-
24783	-	-	-	-	-	-	-	12570	-	-	-
-	-	12348	-	-	-	-	-	-	-	-	-
-	-	14800	-	-	-	-	-	-	-	-	-
1062.6	-	-	-	4843.6	-	-	-	-	-	3782	-
-	-	-	-	-	-	-	-	-	-	-	-
-	-	-	-	-	-	-	-	-	-	-	-
-	-	-	4055	-	-	-	-	-	9963	-	-

C45	C44	C43	C42	C41	C40	C39	C38	C37	C36	C35
0.75	0.74	0.73	0.71	0.7	0.69	0.68	0.67	0.66	0.64	0.62
-	-	-	-	2518.3	-	-	-	-	1706.7	-
-	-	3129	-	1902	-	-	-	-	-	-
-	-	-	1798	-	-	-	900	-	-	982.3
-	-	-	-	-	-	15228	-	-	-	2511.2
-	-	-	-	47531	-	-	-	-	18828	-
-	-	-	-	-	-	-	11323	-	-	34922
-	-	-	-	2002.9	-	-	-	-	-	-
-	-	-	-	-	8567	-	-	-	1934.3	-
-	-	769.8	-	-	1166	-	-	-	-	2432.6
-	-	-	-	2546.3	-	-	-	-	-	-
-	1983	-	-	967	-	-	-	1233	-	-
-	-	-	-	1556.5	-	-	-	965.5	-	861.1
-	-	-	-	2047.2	-	-	-	-	1974.9	-
1446.1	-	-	2198	-	-	-	-	-	1323.9	-
-	-	-	-	-	1483	-	-	-	1300	-
-	-	-	-	-	-	-	66994	-	-	-
12712	-	-	-	-	-	53910	-	-	-	96243
-	-	-	-	1182.6	-	-	-	-	-	-
-	-	-	6747	-	-	-	5965.8	-	12531	-
-	5172	-	-	-	-	-	-	-	23174	-
-	-	-	-	1587.3	-	-	1474.7	-	-	-
-	-	-	-	-	-	-	-	-	-	-
-	-	-	5936	-	-	-	-	6526	-	14997
-	-	-	2657	-	-	-	2769.5	-	-	-

**Total Number of Metabolites**	C56	C55	C54	C53	C52	C51	C50	C49	C48	C47	C46
	0.96	0.88	0.87	0.86	0.85	0.84	0.83	0.81	0.79	0.77	0.76
**10**	-	-	4081	-	-	-	-	0	0	-	867
**12**	-	-	4551	-	-	-	-	-	-	-	-
**9**	-	-	4644	-	-	-	-	-	-	-	-
**6**	437	-	-	-	2464.3	-	-	-	-	-	-
**9**	-	-	3747	-	-	-	-	2764.5	-	-	-
**6**	-	-	-	2840	-	-	-	-	-	-	-
**5**	-	-	-	-	1852.3	-	-	-	-	-	-
**6**	-	-	-	3411.7	-	-	-	-	-	-	-
**12**	-	-	-	3048.9	-	-	-	-	1454	-	-
**3**	-	-	-	-	-	1899	-	-	-	-	-
**5**	-	-	-	2989.3	-	-	-	-	-	-	-
**6**	-	-	-	2478.5	-	-	-	-	-	-	-
**5**	-	-	-	-	-	1963	-	-	-	-	-
**5**	-	-	-	2434.8	-	-	-	-	-	-	-
**9**	-	-	-	2774.5	-	-	-	-	-	-	-
**5**	-	-	-	-	-	-	2664	-	-	-	-
**4**	-	-	-	-	3066.5	-	-	-	-	-	-
**5**	-	-	-	3049	-	-	-	-	-	-	-
**10**	-	-	-	-	10194	-	-	10389	-	-	-
**10**	-	-	-	10595	-	-	-	10880	-	-	-
**10**	-	3559	-	-	-	-	-	-	-	-	-
**2**	-	-	-	7263.6	-	-	-	-	-	-	10281
**9**	-	-	8526	-	-	-	-	-	-	3295	-
**8**	-	-	-	-	-	-	-	-	5588	-	-
